# Isoform analysis of heterozygous putative splicing variants at the allele level using nanopore long-read sequencing

**DOI:** 10.1038/s41598-025-14566-z

**Published:** 2025-08-08

**Authors:** Kokoro Ozaki, Takashi Irioka, Shohei Noma, Akira Machida, Moe Fukunaga, Tatsuro Murano, Chitose Takahashi, Michihira Tagami, Tsugumi Kawashima, Tomoko Hirata, Yuki Yasuoka, Hiroya Kuwahara, Toshiyuki Araki, Ken Yagi, Hidehiro Mizusawa, Kinya Ishikawa, Yasushi Okazaki, Takanori Yokota

**Affiliations:** 1 Laboratory for Comprehensive Genomic Analysis, Riken Center for Integrative Medical Sciences, 1-7-22 Suehiro-cho, Tsurumi, Yokohama, 230-0045 Kanagawa Japan; 2 Department of Neurology and Neurological Science, Graduate School of Medical and Dental Sciences, Institute of Science Tokyo, 1-5-45 Yushima, Bunkyo, 113-8519 Tokyo Japan; 3 Department of Peripheral Nervous System Research, National Institute of Neuroscience, National Center of Neurology and Psychiatry, 4-1-1 Ogawa-Higashi, Kodaira, 187-8502 Tokyo Japan; 4 Diagnostics and Therapeutics of Intractable Diseases, Intractable Disease Research Center, Graduate School of Medicine, Juntendo University, 2-1-1 Hongo, Bunkyo, 113-8421 Tokyo Japan; 5 Department of Neurology, Yokosuka Kyosai Hospital, 1-16 Yonegahama dori, Yokosuka, 238-8558 Kanagawa Japan; 6 Department of Neurology, Tsuchiura Kyodo General Hospital, 4-1-1 Otsuno, Tsuchiura, 300-0028 Ibaraki Japan; 7 National Center of Neurology and Psychiatry, 4-1-1 Ogawa-Higashi, Kodaira, 187-8551 Tokyo Japan; 8 Department of Personalized Genomic Medicine for Health, Graduate School of Medical and Dental Sciences, Institute of Science Tokyo, 1-5-45 Yushima, Bunkyo, 113-8519 Tokyo Japan

**Keywords:** Computational biology and bioinformatics, Genetics, Neurology

## Abstract

One of the challenges in clinical genetics for rare diseases and personalized medicine is evaluating isoform alterations arising from heterozygous putative splicing variants at the allele level. Our aim was to analyze these variants by dividing cDNA or direct RNA nanopore long reads into two alleles, referencing whole-genome sequencing data containing allele-informative single nucleotide variants and then comparing the allele-separated reads using Full-Length Alternative Isoform analysis of RNA (FLAIR), a previously published bioinformatics tool for isoform analysis. In this study, we developed an allele-separative bioinformatics pipeline and described its performance. We applied our pipeline to previously published nanopore direct RNA sequencing data, as well as 5’ cap-trapping full-length cDNA nanopore sequencing (CTR-seq) data from blood samples of three individuals. We successfully identified heterozygous splicing variants associated with significant isoform differences between alleles. Furthermore, we uncovered the effects of a novel pathogenic splicing variant in *PYGM* on isoforms in a compound-heterozygous case of McArdle disease using nanopore cDNA amplicon and targeted genomic sequencing. This study demonstrates the utility of nanopore long-read sequencing for isoform analysis at the allele level, providing a valuable approach to evaluating the direct consequences of heterozygous splicing variants in individuals.

**Supplementary Information:**

The online version contains supplementary material available at 10.1038/s41598-025-14566-z.

## Introduction

One of the challenges in clinical genetics for rare diseases, especially in the era of personalized medicine, is assessing the effect of putative splicing variants on isoform expression in heterozygotes at the allele level. The effects of heterozygous variants are often diluted in diploid humans, making them difficult to detect, particularly when transcripts from the variant-carrying allele are subject to degradation mechanisms frequently associated with pathogenic splicing variants^1^.Therefore, it would be advantageous to computationally divide transcriptome or target transcript sequencing reads into two alleles and identify or evaluate splicing variants associated with statistically significant differences in isoforms expression between alleles.

Although assigning each splicing event to a specific allele presents a challenge, long-read sequencing technologies, such as those from Oxford Nanopore and PacBio, provide a promising opportunity to address this issue. These technologies offer more direct insights into isoform expression and haplotype information by spanning multiple variants within transcripts or the genome^2,3^. Several bioinformatics tools, such as Full-Length Alternative Isoform analysis of RNA (FLAIR) and StringTie2, have been developed to facilitate isoform evaluation from nanopore sequencing data^4,5^.

In this study, we present a concise pipeline, namely “Isoform analysis of heterozygous putative splicing variants at the allele level using nanopore long-read sequencing (ISAHESVAL-NL)”, that applies FLAIR to assess the effect of heterozygous putative splicing variants on isoform differences between two alleles. We analyzed previously published direct RNA sequencing nanopore long-read data^6,7^as well as 5’ capped full-length cDNA nanopore sequencing (Cap Trap RNA-seq, abbreviated as CTR-seq)^8^ data from three individuals. In our pipeline, long reads were separated into two alleles if the read covered an allele-informative heterozygous single nucleotide variant (SNV). In rare cases, splicing variants located on the long read (primarily exonic variants) were haplotyped with other allele-informative SNVs, allowing for the assignment of reads to distinct alleles. Our pipeline successfully identified putative splicing variants associated with statistically significant isoform differences between alleles, based on genome-wide predictions of heterozygous splicing variants.

Additionally, we illustrated allele-specific aberrant isoform expression to show the direct consequences of a heterozygous pathogenic splicing variant in *PYGM*, the causative gene of McArdle disease (glycogen storage disease type V)^9–11^. This was achieved using nanopore cDNA amplicon and targeted genomic sequencing with Clustered Regularly Interspaced Short Palindromic Repeats (CRISPR)/Cas9 enrichment^12^. This study demonstrates the utility of nanopore long-read isoform analysis at the allele level in clinical genetics and personalized genomics, particularly when combined with genomic data.

## Results

### A concise pipeline for allele-discriminative isoform evaluation

**Fig. 1:**
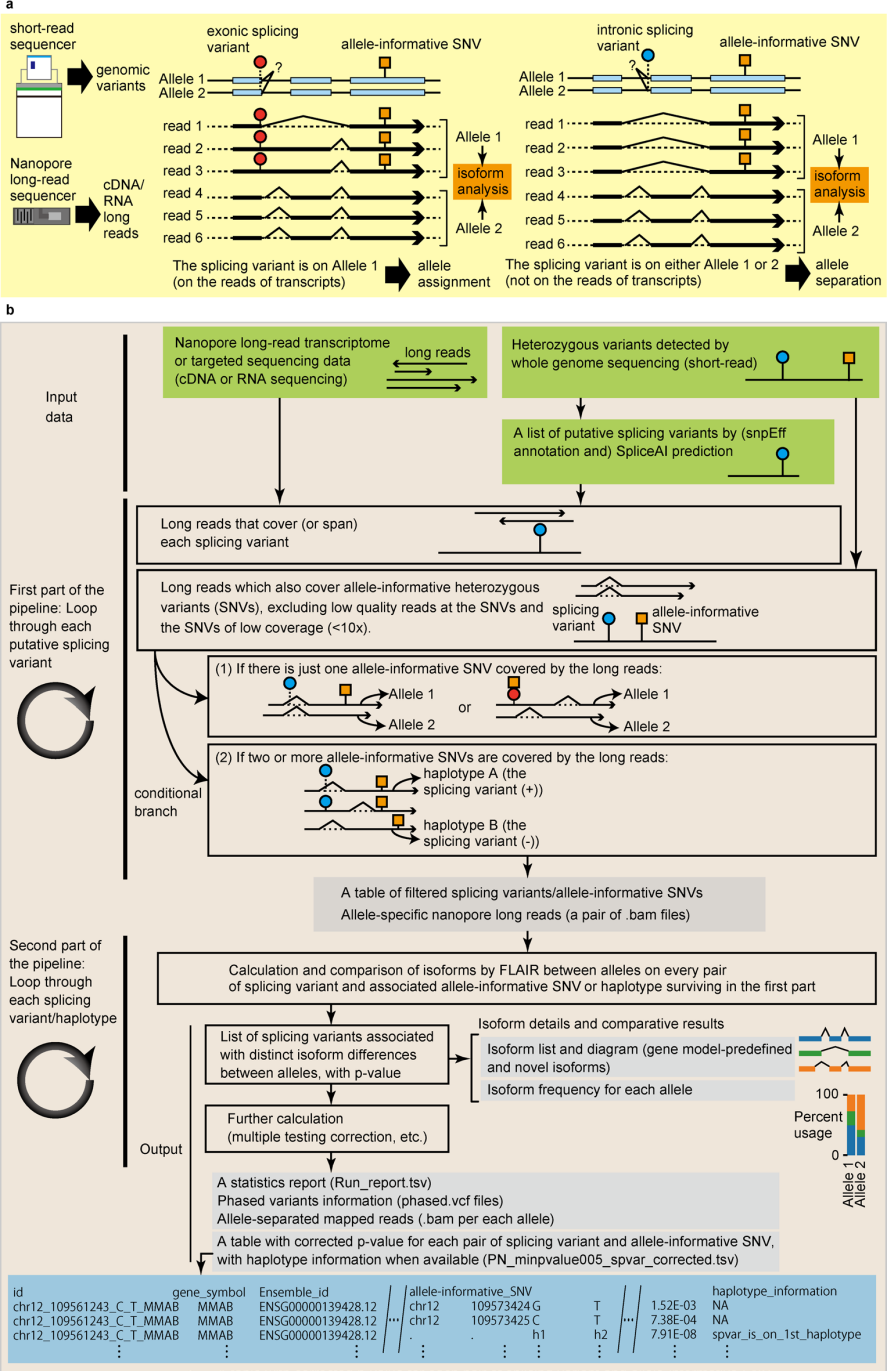
Pipeline for isoform analysis of heterozygous putative splicing variants at the allele level using nanopore long-read sequencing. (**a**) The principle of processing splicing variants and long-reads with respect to alleles in this pipeline. We predicted putative splicing variants based on short-read whole-genome sequencing data. We divided nanopore long reads obtained through direct RNA or cDNA sequencing into two alleles (“Allele 1” or “Allele 2”) based on allele-informative SNVs (orange square pinned to Allele 1). Depending on whether the splicing variant is on the reads of transcripts (for example, exonic splicing variant (red circle pinned to Allele 1)) or not (intronic splicing variant, blue circle, which does not have information on which allele it belongs to), allele assignment (to define the allele which the splicing variant is associated with) or allele separation (to divide long-reads into two alleles based on allele-informative SNV), respectively, was conducted. Subsequently, allelic difference and statistics of isoforms were calculated per splicing variant and allele-informative SNV pair. (**b**) The structure of the pipeline. The Inputs to the pipeline are long-reads, splicing variants, and allele-informative SNVs (green blocks in “Input data” section). In the first part of the pipeline, for each putative splicing variant, we identified nanopore long reads spanning the variant and searched for candidate allele-informative SNVs that provide allelic information to the long reads. After removing reads with low base quality at the allele-informative SNVs and the SNVs with low coverage, we collected sets of nanopore reads separated by allele-informative SNVs, the associated putative splicing variant, and the allele-informative SNVs. We permitted long reads that satisfy one of two criteria: (1) they cover a single allele‑informative SNV—including splicing variants that simultaneously act as allele‑informative SNVs (denoted by the orange square and red circle at the same coordinate)—or (2) they cover multiple allele‑informative SNVs. In the second part of the pipeline, FLAIR is implemented for each set to calculate and visualize isoform lists, and to derive comparative statistics between alleles.

To analyze isoforms at the allele level using nanopore long reads, we developed a pipeline (Fig. 1a and b) that utilizes a recently developed bioinformatics tool called FLAIR (Full-Length Alternative Isoform analysis of RNA). FLAIR calculates and depicts isoforms from nanopore long reads aligned to the reference genome and gene models^4^. Our pipeline consists of two main parts.

The first part of the pipeline processes input data into a summary table and allele-separated .bam (read) files after several filtering steps. Specifically, this part of the pipeline requires three input files: (1) A list of heterozygous putative splicing variants to be evaluated, annotated with snpEff^13^ on whole-genome sequencing data and added with the result of splicing site predictions by SpliceAI^14^(2) A list of heterozygous variants used for discriminating between the two alleles, which we refer to as “candidate allele-informative single nucleotide variants” (candidate allele-informative SNVs or simply allele-informative SNVs), and (3) Long-read cDNA or direct RNA sequencing data mapped against the reference genome (GRCh38). The lists of heterozygous putative splicing variants in this study were prepared with snpEff annotation and SpliceAI predictions. Because snpEff can be used to markedly narrow down variants to be evaluated by SpliceAI, it is useful especially for a quick check. For more comprehensive listing of splicing variants, annotation with snpEff was not used in narrowing down and only delta score by SpliceAI was used. Delta score in the results of SpliceAI is defined as the maximal probability of splicing alteration within the window (default set as 50 bp from the variant in the version used) by acceptor gain, acceptor loss, donor gain, and donor loss, and ranges from 0 to 1. For example, a variant chr8:129750591 C > T (ENST00000276708.9, c.944-21G > A) in *GSDMC*, was predicted to have delta scores such as (acceptor gain (at -2 bp position) 0.95 | acceptor loss (-21 bp) 0.92 | donor gain (4 bp) 0.00 | donor loss (-43 bp) 0.00), showing an example of high delta score in a splicing acceptor site. In contrast, another variant chr5:168468948T > C (ENST00000265293.9, c.3276-3T > C) in *WWC1*, was predicted to have delta scores such as (acceptor gain 0.27 | acceptor loss 0.00 | donor gain 0.00 | donor loss 0.00) at positions (3|-2|37|-38), showing an example of lower delta score as a splicing acceptor site and score 0 as a splicing donor site. Thus, each variant within the defined gene regions in the genome has delta scores as a result of the calculation. We applied different delta score thresholds for SpliceAI (0.2, 0.5, and 0.8) to generate lists of splicing variants with high recall (low precision), moderate recall/precision, and high precision (low recall), respectively. For each splicing variant, we filtered long reads that spanned the coordinate of the splicing variant and checked whether 10 or more long reads contained a base (A/T/G/C) and had base quality ≥ 10 (90.0% reliability) at each candidate allele-informative SNV using jvarkit^15^.

Our pipeline automatically counts available numbers of allele-informative SNVs, including splicing variant itself in case of exonic splicing variant or intronic variant expressed when intron retention occurred. If two or more variants are available and covered by long reads, then whatshap^16^a bioinformatics tool for haplotype calculation, assigns haplotypes. A result table “PN_minpvalue005_spvar_corrected.tsv” contains information on haplotypes. When the haplotype is calculated for the splicing variant and allele-informative SNVs, result folder contains phased variant call file (splicing variant-associated phased.vcf.gz), in which genotype is added by whatshap, such as “0|1” or “1|0” for each variant included. By looking at this information, one can see phases of each variant within this haplotype block. When haplotype information is not created or does not harbor the splicing variant (such as in case of intronic splicing variant) in the phased.vcf.gz file, one can still see separation of long-reads and how the two alleles of the allele-informative SNV are associated with separated isoforms (Fig. 1a, right panel). In the case of Patient2 later described, independent genomic analysis enabled haplotyping of a novel intronic splicing variant with the known missense variant outside of the cDNA amplicon. Interpreting the result of our pipeline in combination with genomic analysis thus can be used to help understand allele-specific isoform changes. The output of this first part of the pipeline consists of paired .bam files for each allele, splicing variant, and allele-informative SNV or haplotype, which are preserved for further calculation in the second part.

In the second part of the pipeline, these paired .bam files for alleles and associated gene names are used to calculate isoform differences between alleles using FLAIR. Both parts of the pipeline employ parallelization for faster computation (details are provided in the Methods section). The final outputs include PN_minpvalue005_spvar_corrected.tsv (a table with each line consisting of splicing variant, considered allele-informative SNV (or haplotype), long-read coverage for Ref and Alt allele of allele-informative SNV, lowest p-value for isoform changes by FLAIR, and haplotype information) (the bottom blue block in Fig. 1b), isoform classification diagrams, and isoform frequency data for each .bam file pair corresponding to each splicing variant and allele-informative SNV set. The detailed information for preparation of input files and output folders and files are described in Supplementary Note 5 and 6.

### Isoform evaluation at the allele level for nanopore direct RNA sequencing

We first tested the pipeline on previously published transcriptome data from nanopore direct RNA sequencing of GM12878^6^ (a HapMap project sample widely used as a standard human lymphoblastoid cell line), combined with short-read whole-genome sequencing data. From the whole-genome sequencing data, we predicted 401 heterozygous putative splicing variants using SpliceAI with a delta score threshold of 0.5 (moderate sensitivity and precision) without narrowing down variants by snpEff annotation. When this list of heterozygous putative splicing variants was used as input for the pipeline, we filtered 144 splicing variants which had at least one associated allele-informative SNV or haplotype and proceeded with FLAIR isoform analysis in the second part of the pipeline.

**Fig. 2:**
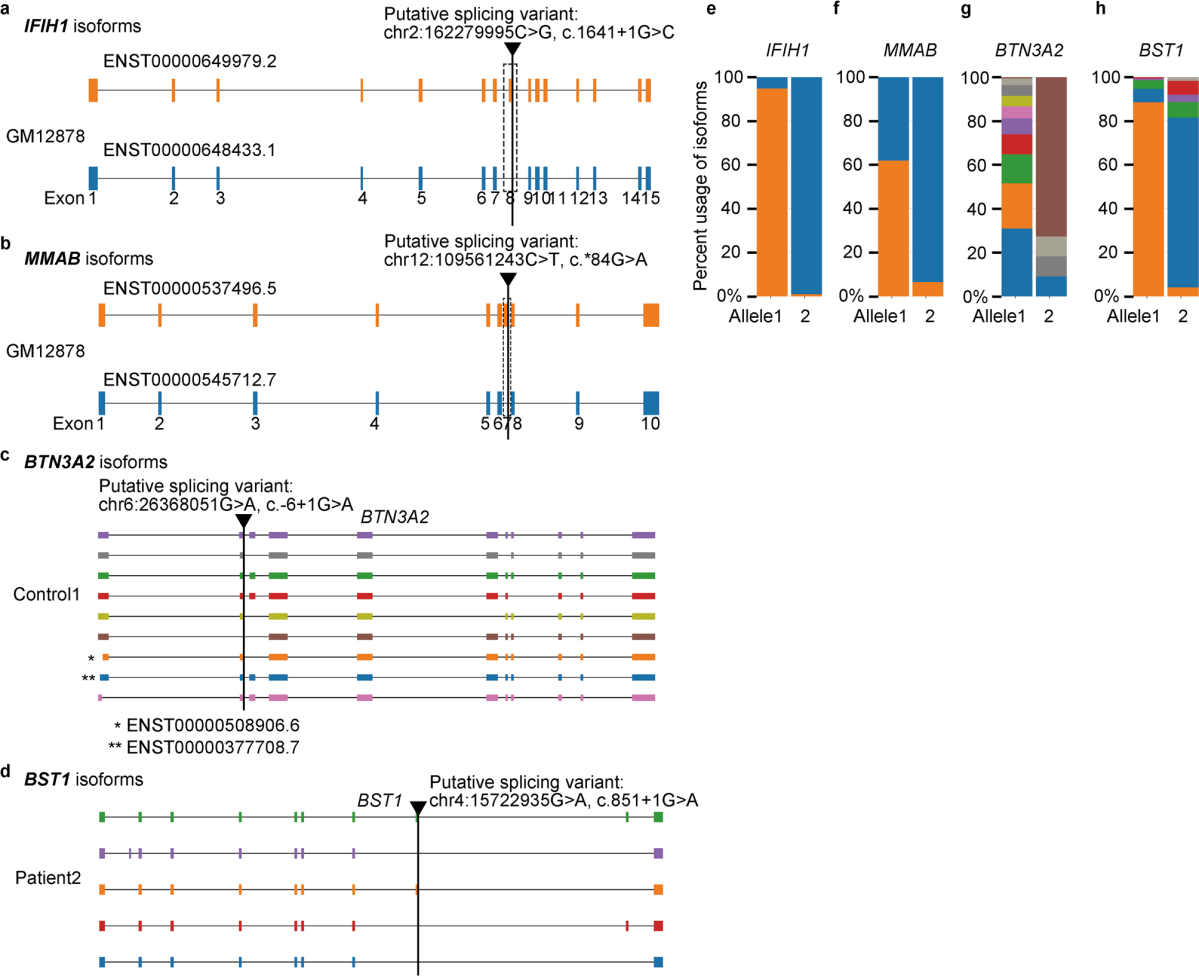
Identified splicing variants and allelic isoform differences in nanopore direct RNA sequencing and 5’ cap-trapping full-length cDNA sequencing (CTR-seq). (**a-d**) Diagrams of calculated isoforms, and bar-graph their respective isoform usage (**e-h**). Each isoform model, signified as a track in diagram, was supported by multiple long-reads along the parameters in FLAIR implementation. Isoforms labeled with Ensemble id (“ENST + number”) were those identified by FLAIR as registered transcript. Other isoforms without Ensemble id represent those not identified as registered in the gene model but exist in the sample. *IFIH1* (**a** and **e**) and *MMAB* (**b** and **f**) are heterozygous splicing variants with associated allelic isoform differences, as calculated in the pipeline from direct RNA sequencing data. These variants were among previously reported loci with allelic isoform differences in the original article. The allelic isoform frequency differences are notable at the loci (**e** and **f**). The same colors between the diagram and corresponding isoform usage bar graph represent the same isoform. For instance, exon 8 is absent (highlighted by the dashed line in **a**) in the blue-colored isoform (ENST00000648433.1), which is almost exclusively expressed in one allele (e). **(c**,** d**,** g**,** and h**) Identified splicing variants using CTR-seq, chr6:26368051G > A (c.-6 + 1G > A; NM_007047.5; *BTN3A2*) in Control1 (**c** and **g**) and chr4:15722935G > A (c.851 + 1G > A; NM_004334.3; *BST1*) in Patient2 (**d** and **h**), which were both associated with skipping of the neighboring exon in one of the alleles, were shown (dark-brown colored transcript in (**c**) and (**g**), and blue colored transcript in (**d**) and (**h**), respectively).

As a result, we identified 14 heterozygous putative splicing variants with statistically significant (p-value, Bonferroni corrected < 0.05) isoform differences between alleles, including *IFIH1*, a gene noted for having markedly different isoform composition between alleles in the original study^6^ (Fig. 2a and e, and Supplementary Table 1). In addition to the splicing variant in *IFIH1*, another heterozygous splicing variant in *MMAB*, which was also among the 34 genes found to have a unique isoform per allele in the published study, was shown to be associated with isoform changes (Fig. 2b and f, and Supplementary Table 1).

We then tested the pipeline on the Nanopore long-read direct RNA sequencing data from cancer cell lines K562 (leukemia) and HepG2 (hepatocellular carcinoma) from The Singapore Oxford Nanopore Expression Project (SG-NEx)^7^combined with short-read whole-genome sequencing data (see details in the Method section). We predicted 70 and 101 heterozygous putative splicing variants, respectively in K562 and HepG2, using SpliceAI with a delta score threshold of 0.5 after narrowing down variants with snpEff annotation to only variants associated with splicing. As a result of our pipeline implemented with the same parameters as in GM12878, we identified two and two heterozygous putative splicing variants with statistically significant isoform differences between alleles, respectively in K562 and HepG2 (Supplementary Tables 2 and 3). The two genes, *GIPC1* and *RIPK2*, where the two heterozygous splicing variants with isoform changes in K562 were found, of interest, are involved in tumor growth or invasion^17,18^ while the two genes, *ULK3* and *XRCC4*, where the two heterozygous splicing variants with isoform changes in HepG2 were found, are known to be elevated in squamous cell carcinoma and associated with cancer susceptibility^19,20^.

### Isoform evaluation at the allele level for full-length nanopore cDNA transcriptome

To further test our pipeline, we conducted full-length 5’ cap-trapped and poly-A captured whole transcriptome nanopore long-read sequencing (CTR-seq). This approach, proven effective in a previous study^8^ was applied to total blood RNA from three subjects: a healthy control (Control1), a patient with undiagnosed cerebellar ataxia (Patient1), and a patient with biopsy-proven McArdle disease who harbors both a novel splicing variant and a known missense variant (Patient2, the proband (II-1) in Supplementary Fig. 1a, b, and c). The clinical characteristics of the three subjects, along with detailed information about Patient2, are provided in Supplementary Note 1 and 3 and discussed further in the next section.

The sequencing statistics, including read number and quality, were comparable across the samples (Supplementary Table 4), but the average read length was relatively shorter in Patient1 (median read length: Control1, 1608 bp; Patient1, 1317 bp; Patient2, 1721 bp), possibly due to differences in sample quality. The bioinformatics pipeline was implemented as described earlier. The number of heterozygous putative splicing variants as inputs, predicted by SpliceAI with delta score 0.5 or higher, were 420 (Control1), 389 (Patient1), and 457 (Patient2). Of these, 143, 100, and 152 cumulative combinations of filtered splicing variants and associated allele-informative SNV(s) or haplotypes were further analyzed for isoform differences.

As a result, 25, 7, and 15 unique splicing variants, respectively, were found to have significantly distinct isoform compositions between alleles (p-value, Bonferroni correction < 0.05) within each subject (Supplementary Tables 5, 6, and 7). For example, heterozygous splicing variants such as chr6:26368051G > A (c.-6 + 1G > A; NM_007047.5; *BTN3A2*) in Control1 and chr4:15722935G > A (c.851 + 1G > A; NM_004334.3; *BST1*) in Patient2 were associated with skipping of the neighboring exon in one of the alleles (Fig. 2c, d, g, and h).

As for the 7 splicing variants identified in Patient1 with cerebellar ataxia (Supplementary Table 6), there is not a gene which is directly linked to neurodegeneration other than *XRCC4*, which was reported to cause microcephaly and progressive ataxia^21^. However, the allele frequency of the splicing variant in *XRCC4* is high (Mean Allele Frequency of 0.7011) in East Asian population in gnomAD^22^ and is not considered to be causative for a rare disease. Although there might be another unidentified genetic variant that is responsible for Patient1, other underlying causes, such as epigenetic modifications, might also be possible.

And among 15 splicing variants identified in Patient2 with McArdle disease (Supplementary Table 7), no variants were found in *PYGM*, which is the causative gene for McArdle disease. A variant (chr1:179889309G > A) in *TOR1AIP1*, which is known to cause limb-girdle muscular dystrophy^23^ was found, but it has very high allele frequency (0.6240) in global population and unlikely to cause such rare disease. No other variant that can account for muscular pathology in this patient was found.

### Parameter consideration for the pipeline

**Fig. 3:**
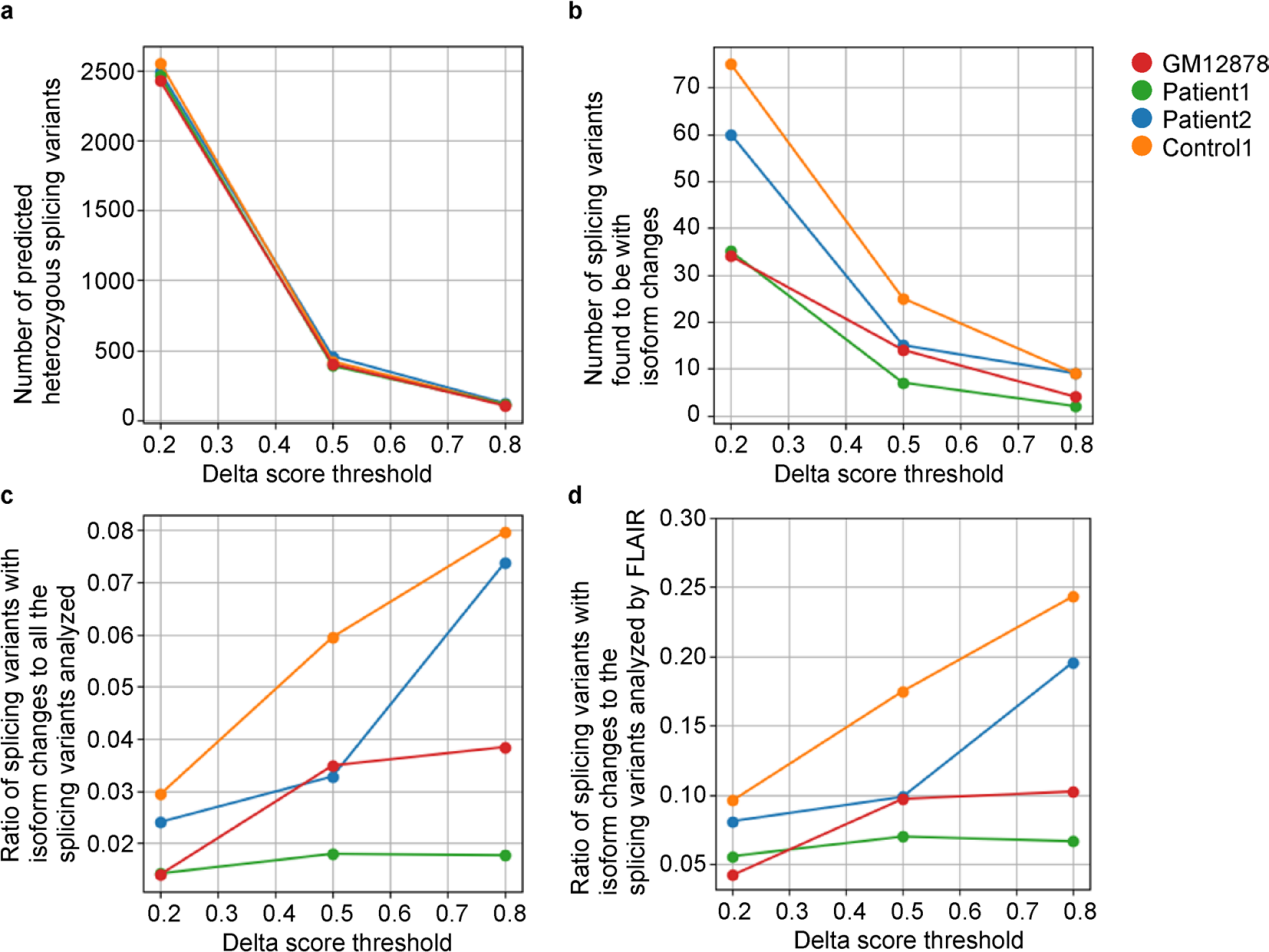
Parameter consideration for the pipeline. (**a**) The number of heterozygous splicing variants predicted by SpliceAI. (**b**) The number of splicing variants calculated to be with statistically significant isoform changes. (**c**) The ratio of splicing variants with isoform changes to all splicing variants analyzed in the first part of the pipeline (Fig. 1). (**d**) The ratio of splicing variants with isoform changes to all splicing variants analyzed by FLAIR in the second part of the pipeline. Each data series for GM12878, Patient1, Patient2, and Control1 is represented by red, green, cyan, and orange dots, respectively.

To further optimize the parameters of the pipeline, we examined how they affect the analysis results for nanopore direct RNA sequencing (GM12878) and CTR-seq (Control1, Patient1, and Patient2). First, we explored how the delta score thresholds for SpliceAI, ranging from 0.2 to 0.8, influenced the number of predicted heterozygous splicing variants for each subject. As described by the SpliceAI developers, the confidence in splicing variant prediction increases as the delta score increases: 0.2 for high recall (low precision), 0.5 (recommended threshold) for moderate sensitivity and precision, and 0.8 for high precision (low recall). We observed an approximately five-fold reduction in the number of predicted heterozygous splicing variants when increasing the delta score threshold from 0.2 to 0.5, with a milder reduction when increasing the threshold from 0.5 to 0.8 (Fig. 3a). This means that more splicing variants are put in the pipeline if the lower stringent delta score threshold is used. We observed that more heterozygous splicing variants which were finally found to be associated with statistically significant changes (corrected p-value) remained when lower delta score threshold was used (Fig. 3b).

Next, we investigated how the delta score threshold affects the likelihood that the tested splicing variants are associated with statistically significant isoform changes between alleles. We found that heterozygous splicing variants predicted with higher delta score thresholds tended to be more likely to be associated with statistically significant changes (corrected p-value < 0.05) in isoform content, as determined by FLAIR analysis (Fig. 3c). This trend was consistent between the delta scores of 0.2 and 0.5 across all samples, but was not observed between 0.5 and 0.8 in Patient1. We observed this trend both in the ratio of splicing variants with statistically significant isoform changes to all splicing variants analyzed in the first part of the pipeline (Fig. 1), as well as in the ratio of splicing variants with significant isoform changes to those filtered and analyzed by FLAIR in the second part of the pipeline (Fig. 3d). Thus, more predicted heterozygous splicing variants are to be subjected to evaluation in the pipeline when lower threshold for delta score is used, and result in finding increasing number of splicing variants with significant isoform changes, but at a lower rate.

We also examined the distribution of read lengths covering splicing variants with significant isoform changes between alleles, comparing it to the distribution of read lengths covering splicing variants that were not filtered out in the first part but did not show significant isoform changes in the FLAIR analysis. No consistent or clear pattern was observed across four samples (GM12878, Control1, Patient1, and Patient2) regarding whether longer reads were more likely to contribute to splicing variants with significant isoform changes (Supplementary Fig. 2). Given that the read length is inherently limited by the cDNA length, the trivial differences between the reads covering the splicing variants with significant isoform changes and those without may not be impactful (Supplementary Fig. 2), as long as the read spans the splicing variant and the altered exon-intron junction.

### Isoform evaluation of *PYGM* at the allele level by nanopore cDNA amplicon sequencing in a patient with McArdle disease

Sanger sequencing of *PYGM* in Patient2 revealed a novel intronic variant (*PYGM*, NM_005609.3; c.1519-3T > G, chr11:64752507 A > C (GRCh38)) within intron 12–13, located 3 bp upstream of the start of exon 13, as well as a known pathogenic missense variant (c.347T > C, chr11:64758514 A > G (GRCh38), p.Leu116Pro) in exon 3 (Supplementary Fig. 1b). These two variants were confirmed to be in a compound heterozygous state, using CRISPR/Cas9 enriched targeted nanopore sequencing^12^ (Supplementary Fig. 1d and e). SpliceAI predicted that the novel intronic variant c.1519-3T > G was highly likely to affect splicing (delta scores: acceptor gain 0.98, acceptor loss 0.98, donor gain 0, donor loss 0), and it also had a CADD PHRED score of 23.6, above the commonly used threshold of 20 (top1%)^24^. A muscle biopsy specimen analyzed via reverse transcription-polymerase chain reaction (RT-PCR) and subsequent cloning further supported the presence of mis-spliced transcripts (Supplementary Fig. 3).

**Fig. 4:**
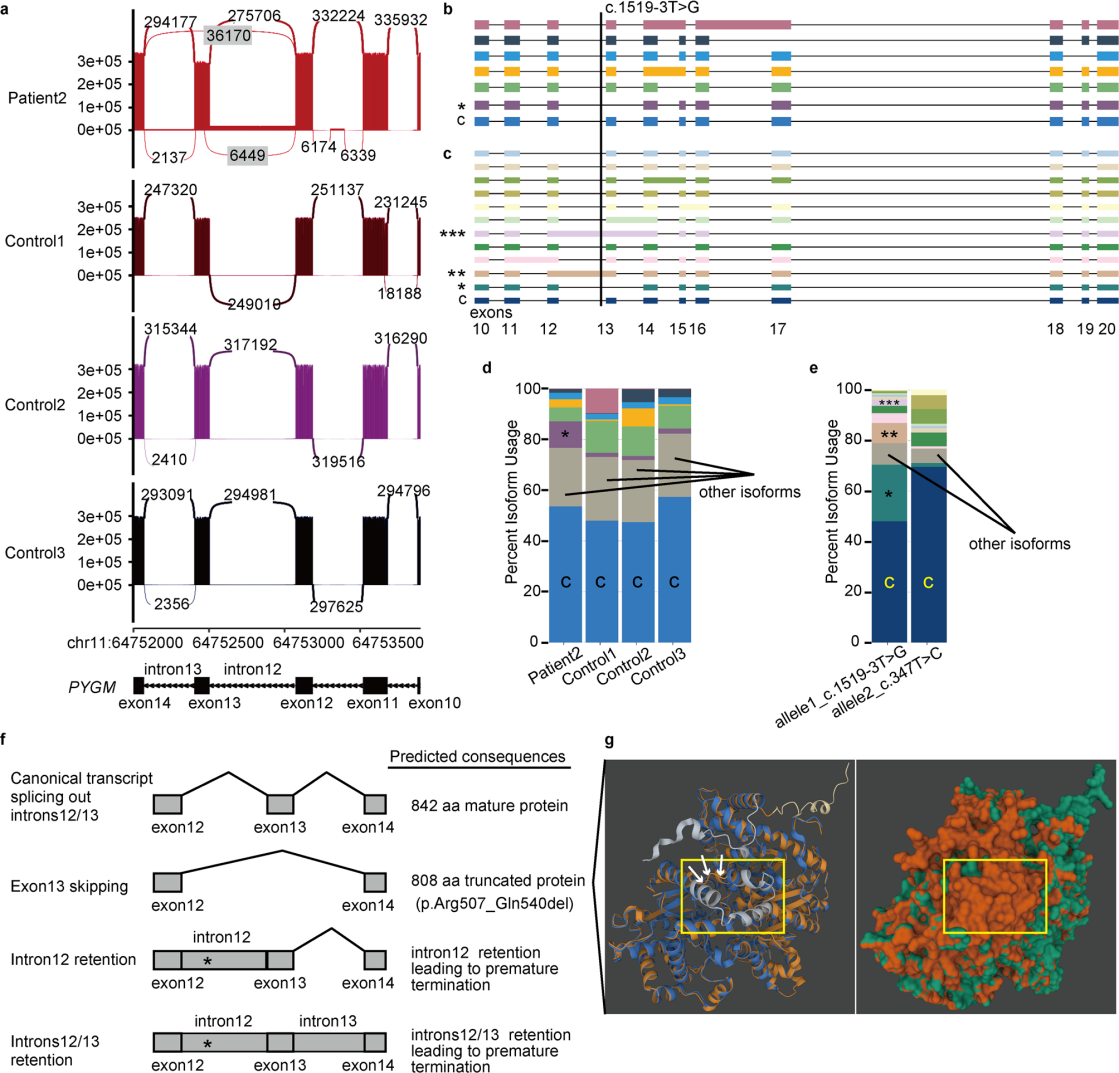
Isoform evaluation of *PYGM* at the allele level by nanopore cDNA amplicon sequencing in a patient with McArdle disease. (**a**) Sashimi plot of nanopore cDNA amplicon sequencing in Patient2 (with a putative splicing variant and a missense variant) and three controls. While splicing abnormalities, such as intron 12 retention, were observed in some reads, the complete isoform content in Patient2 remains unclear. (**b** and **d**) Comparison of isoform contents between samples without allele separation or assignment, showing the exon 13 skipping isoform (marked by an asterisk in d) in Patient 2. The letter C denotes the canonical transcript. (**c** and **e**) Allele-assigned isoform analysis showed that the allele with the c.1519-3T > C splicing variant led to various splicing abnormalities, including exon 13 skipping (asterisk), intron 12 retention (double asterisks), and retention of both introns 12 and 13 (triple asterisks). (**f**) Predicted molecular consequences of the c.1519-3T > C splicing variant in Patient2. Exon 13 skipping is predicted to produce a truncated protein, while retention of intron 12 or both introns 12 and 13 is expected to result in premature termination (asterisk signifying a stop codon which terminates intron12 translation). (**g**) Pairwise structural alignment of AlphaFold2-predicted myophosphorylase protein, which lacks the R507_Q540 region (34 amino acids) due to exon 13 skipping caused by the novel splicing variant, compared to the wild-type protein. Left: Cartoon structure showing the wild-type protein in blue and the mutant protein in brown. Right: Surface structure showing the wild-type protein in orange and the mutant protein in green. Yellow rectangles indicate the R507_Q540 portion of the wild-type protein (in silver) and the truncated region in the mutant protein (arrows).

The *PYGM* variants in Patient2 did not pass the criteria of the pipeline for CTR-seq data, due to insufficient read coverage for the two variants. There were only two reads in the novel splicing variant of *PYGM*, and even when we implemented the pipeline with the lowest parameters (1 read for allele-informative SNV, and 1 read for FLAIR collapse step), it still could not catch the isoform changes associated with the novel splicing variant, because of too few reads. This limitation prompted us to further analyze *PYGM* transcripts using cDNA amplicon sequencing to detect isoform changes more sensitively. To overcome the limited availability of muscle specimen, we analyzed whole blood RNA samples using nanopore long-read sequencing of a 2 kb cDNA amplicon encompassing exons 9–20 of *PYGM*. The sashimi plot revealed exon 13 skipping and intron 12 retention, but the overall picture of aberrant splicing and isoform changes remained unclear (Fig. 4a).

When comparing *PYGM* isoforms between Patient2 and three controls (clinical details in Supplementary Note 2) without separating/assigning alleles, exon13-skipped isoform (asterisk in Fig. 4b and d) was detected in 10.4% of the reads in the patient. Further evaluation of isoforms at the allele level within Patient2 more clearly showed detailed isoform changes: A part of cDNA amplicon reads covered the novel splicing variant by intron retention and other two allele-informative SNVs, and made it possible to assign the reads into the two alleles. Transcripts from the allele harboring the novel splicing variant (allele1_c.1519-3T > G in Fig. 4e) tended to produce exon13 skipping (22.3% of the reads, shown as single asterisk in Fig. 4c and e) or retention of intron12 (7.9%, double-asterisk), or both introns 12 and 13 (2.4%, triple-asterisk). The latter two cases were predicted to result in out-of-frame insertions of 568–899 bp, leading to premature termination of translation (Fig. 4c, e, and f). Exon13 skipping was predicted to affect the superficial structure of the PYGM protein, as modeled by alphafold2^25^ (Fig. 4g).

### Calculation time

As for calculation time on the pipeline, it was 160.4, 22.6, 3.9 h, respectively, for delta score thresholds of 0.2, 0.5, and 0.8 on the direct RNAseq data of GM12878. Also, when viewed from user + sys CPU time, they were 1690.9, 260.2, 36.5 h, respectively (Supplementary Table 8). Thus, the resulting computation time increased as the number of input variants increased in lower delta score threshold.

Therefore, the real time was achieved by parallelization using GNU-parallel^26^ in the first half of the pipeline and GNU-parallel plus FLAIR (internal parallelization) in the second half, on average about 10 cores working in parallel, when we set number of GNU-parallel threads as 10 in the first half and 6 in the second half.

## Discussion

We presented a bioinformatics pipeline designed to evaluate direct consequences of heterozygous putative splicing variants through nanopore direct RNA and cDNA sequencing in combination with genomic data. As demonstrated in this study, our pipeline successfully identifies heterozygous putative splicing variants associated with significant isoform differences between alleles. It also allows for the illustration of isoform content per allele, with either allelic separation or allelic assignment, depending on the genomic information available for each subject.

This method can evaluate a list of heterozygous putative splicing variants within a single individual by integrating nanopore direct RNA sequencing or cDNA sequencing with genomic data, preferably whole-genome sequencing or phased variant data from long-read sequencing. As such, the pipeline is well-suited for patients with rare diseases or other clinical contexts in personalized medicine. Notably, it offers a quick assessment of a single rare disease sample without needing control samples from healthy individuals or multiple patients with the same disease and variant, which is especially useful when working with cells, tissues, or organs that are not readily available from controls.

Given that transcripts from pathogenic splicing variants are often degraded, the transcript level of the gene of interest may be low and detectable only through amplification in clinical samples. In such cases, it is often necessary not only to perform transcriptome-wide analyses but also to analyze to focus on targeted, amplified products. As demonstrated, we showed the utility of allele-level isoform analysis in targeted amplicon nanopore long-read sequencing in a patient with McArdle disease.

In optimizing the pipeline’s parameters, we observed a trend: the higher the threshold for delta score in filtering input splicing variants, the higher the ratio of splicing variants with statistically significant isoform changes to the total filtered variants analyzed by FLAIR. This trend aligns with the fact that splicing variants selected by higher delta scores, (i.e., higher precision by SpliceAI) are more likely to include heterozygous splicing variants associated with significant isoform differences between alleles. However, depending on clinical and research needs, it may sometimes be important to capture as many splicing variants as possible, even at the expense of longer calculation times. We recommend use of 0.5 delta threshold for general use and fast sketch of splicing variants, balancing between the output data extensiveness and computing time, while one may use 0.2 delta threshold for evaluating as many as variants in the subjects with rare diseases. Another important parameter in this pipeline is the threshold of base quality score of long-reads at the allele-informative SNV sites. As described above, our default setting for this value is 10, which necessitates base accuracy of 90% or over, a little lower level compared to the mean accuracy level of older R.9.4.1 or R10.3 chemistries (94–96%)^27,28^. Because of recent advancement in read accuracy in Oxford Nanopore (99.1% with R10.4 chemistry)^29^ as well as PacBio HiFi (> 99.5%)^30^ this setting may be changed accordingly. When the default threshold of base quality score 10 is applied, mis-separation can occur at most for 10% of the reads. Although long-reads with erroneous base call other than reference and alternate allele (e.g., A and T if the reference is C and alternate allele is G at the allele-informative SNV) may be removed and the mis-separation rate can be lower, it is still a key point in the pipeline. So, in addition to the cases applying long-reads with higher accuracy, one may want to change this threshold of base quality score at allele-informative SNVs to evaluate minor isoforms with higher stringency. In the analysis of amplicon cDNA sequencing, the minimum base quality score was set to 15.

One more parameter which is also important is number of required supporting reads covering each allele-informative SNV for each splicing variant, which we set as 10 as a default setting. When we consider a few reads are needed to support each isoform in “FLAIR collapse” phase such as “--support 4” setting (in the case four reads are necessary), it is reasonable that two alleles expressing distinct isoforms will have at least more than double the number of the supporting reads per one isoform. Considering that most alleles will express more than two or more isoforms, this level of supporting 10 reads at each allele-informative SNV is very low level of requirement. If long-reads (cDNA or RNA-seq) are less than 10 reads at the site of all the candidate allele-informative SNVs, then the splicing variant is dropped and will not be further analyzed. This threshold is also in parallel with 10 supporting reads set as a threshold for haplotype-aware analysis in nanopore direct RNA sequencing in the previous report^6^. In addition, when we set the parameters at lower level, such as 5 reads support for allele-informative SNVs and flair collapse --support 3, then identified splicing variants with raw p-value < 0.05 increased from 36 to 41 in direct RNA sequencing data in GM12878. However, number of surviving splicing variants with corrected p-value < 0.05, which is needed for correcting such multiple testing, is again 14 variants on this setting. Therefore, splicing variants with remarkable isoform changes endured several setting levels. Again, parameter optimization is important for each samples/dataset starting from this setting.

There are several factors which can skew the result of our pipeline. From a viewpoint of genetic factors, low allele frequency at allele-informative SNVs may be a major problem. Because our pipeline only can separate RNA/cDNA long-reads if any allele-informative SNVs exist, splicing variants which are well-covered by RNA/cDNA long-reads but scarce allele-informative SNVs are underrepresented in the result. Therefore, one should be cautious that potential isoform changes underrepresented by scarcity of surrounding allele-informative SNV may stay undetected. Furthermore, splicing sites which are surrounded by population-specific allele-informative SNVs might be overrepresented in such population. Similarly, distinct haplotype which is prone to affect splicing may be overrepresented if there are concentrated SNVs surrounding the splicing variants.

The limitations of our study include: (1) A limited number of clinical samples, (2) The nanopore long-read sequencing data could benefit from the latest chemistries, such as Oxford Nanopore’s Q20 + or Duplex sequencing chemistry, which are expected to deliver higher read quality, (3) The core part of the scripts is written in Bash, which may be slower compared to fully Python-based scripts with massive parallelization using graphic processing units (GPUs), (4) Notably, the relationship between each splicing variant, the selected long-reads covering the variant, and the isoform changes arising from nearby exon-intron junctions does not establish causality and requires further experimental validation, (5) As to chemistries in Oxford Nanopore long-reads, we used R10.3 chemistry for CTR-seq, while we used R9.4.1 chemistry in amplicon cDNA and CRISPR/Cas9-enriched genomic sequencing. The reported error rate for these chemistries is about 4 to 6% when base-called in HAC or SUP mode^27,28^. Recently, R10.4 chemistry of Nanopore became available, which achieves 99.1% of accuracy^29^. We have neither tested PacBio HiFi long-reads in our pipeline. Because they are of higher base qualities (> 99.5%)^30^ more detailed calculation in isoforms might be possible. At the same time, our pipeline may need further optimization, and (6) Our pipeline may not be advantageous when an abundant number of appropriate control samples are available for comparison, such as heterozygous patients and homozygous wild-type individuals.

We have not tried to identify splicing variants themselves by nanopore direct RNA or cDNA sequencing, because we might miss intronic variants. And because recent technological advancement and cost reduction in long-read sequencing, it would be interesting to assign each changed isoforms to splicing variants (or to the other allele) based on haplotype information which long-read genomic sequencing will provide in a genome-wide manner. Our current version of this pipeline is not applicable for the long-read generated haplotyped variants, future update might be considered.

In summary, we developed a pipeline that evaluates heterozygous splicing variants by dividing nanopore long-reads into two alleles based on allele-informative SNVs. It systematically detects isoform differences related to splicing variants between alleles. This method will be useful for analyzing transcriptome (cDNA and direct RNA sequencing) and targeted amplicon cDNA sequencing data, helping to discover splicing variants and associated isoform changes in rare disease patients.

## Methods

### Participants

A total of five subjects who underwent clinical evaluation and genetic studies at the Department of Neurology and Neurological Science, Institute of Science Tokyo hospital, were included in this study. Genetic analyses were conducted after obtaining written informed consent from all participants. One patient with McArdle disease (referred to as Patient2) underwent a muscle biopsy for pathological diagnosis, which was required as part of clinical practice, with written consent. All subjects, including the three controls, are of Japanese origin. Control1 is the daughter of Patient1. Other patients and controls are unrelated. The age and sex of the subjects are provided in Supplementary Note 1 and 2. This study was approved by the institutional review boards of Institute of Science Tokyo and Riken. All procedures and methods were implemented following the relevant rules/laws and guidelines, and in accordance with the Declaration of Helsinki.

GM12878, a widely used HapMap project sample, is exempt from IRB/human ethics regulations. We downloaded data of GM12878 direct RNA sequencing by Oxford Nanopore long-reads and genome variants obtained by short-read sequencing (Illumina platinum genome) from published or publicly available sources: (https://github.com/nanopore-wgs-consortium/NA12878/blob/master/RNA.md) and (https://hgdownload.soe.ucsc.edu/gbdb/hg38/platinumGenomes/NA12878.vcf.gz).

### Whole-genome sequencing and data processing

A total of 100 ng of genomic DNA extracted from peripheral blood leukocytes was used for library preparation. A short-read (150 bp paired-end) next generation sequencing library was created using the MGIEasy FS DNA Library Prep Kit V2.0 and subsequently sequenced on a DNBSEQ-G400 sequencer (MGItech, China). For the analysis of whole-genome sequencing data, custom in-house scripts were used for demultiplexing, and the DRAGEN^31^ Bio-IT platform (Illumina, USA) were utilized for pre-processing, mapping to GRCh38, and variant calling. More specifically, Field Programmable Gate Array (FPGA) version of DRAGEN germline (v4.0.3) tool (Software version: 07.021.510.3.5.7, Hardware image version: 07.021.510) was used for subjects other than GM12878, who was analyzed with the publicly available illumina Platinum Genome variant data (see details in “Participants” section). DRAGEN was run with default settings with a typical DRAGEN commands (dragen -r reference − 1 read1.fastq − 2 read2.fastq --enable-variant-caller true --vc-emit-ref-confidence GVCF --vc-enable-vcf-output true --output-directory out_dir --output-file-prefix prefix --enable-map-align true --enable-sv true --enable-cnv true --output-format BAM --enable-map-align-output true --enable-bam-indexing true).

Although DRAGEN is optimized for processing reads from illumina sequencers, whole-genome sequencing data from MGITech was also applicable. Comparison of variant calls between whole-genome sequencing data of GM12878 at 30x coverage from illumina HiSeq and MGITech DNBSEQ-G400, both of which downloaded from publicly available sources, gave a highly concordant result, especially for SNVs, with F1 score of 0.9738 (details in Supplementary Note 7 and Supplementary Table 9), while indel calls tend to be unique to each platform (F1 score of 0.9165). This tendency is in line with a previous report on comparison of both platforms in whole-genome sequencing of a subject^32^. For reconstructing headers of fastq files from MGI type to illumina type, bgi2illumina was used (Supplementary Note 7).

### Downloading and processing of publicly available whole-genome sequencing and Oxford nanopore direct RNA sequencing data of cell lines (K562 and HepG2)

Whole-genome sequencing reads data (.fastq) were downloaded from a public database ENCODE^33^ with the following identifiers: ENCFF826SYZ and ENCFF471WSA (K562) and ENCFF256YUY and ENCFF852MGK (HepG2) which were both sequenced by illumina HiSeq X Ten and contributed by Alexander Urban (Stanford) to the database^34^. Reads data were then mapped and variant-called with DRAGEN (SW: 05.121.645.4.0.3, HW: 05.121.645) germline tool (v 4.0.3) as the illumina basespace application with the command-line options such as: “--lic-server https://XXXXXXXXXXXX:YYYYYYYYYYYYYYYYYYYYYYYYYYYYYYYY@license.edicogenome.com --lic-instance-id-location /root/.edico --output_status_file /data/scratch/progress.log --enable-map-align true --enable-duplicate-marking true --enable-map-align-output true --output-format BAM --enable-cnv true --cnv-enable-self-normalization true --cnv-segmentation-mode slm --cnv-enable-ref-calls true --sample-sex auto --enable-bam-indexing true --enable-metrics-json true --json-dataset-type /staging/files/dataset-types/dragen_complete_v031.json --enable-variant-caller true --enable-vcf-compression true --vc-emit-ref-confidence GVCF --vc-enable-vcf-output true --vc-enable-bqd true --output-directory out_dir --intermediate-results-dir /data/scratch/intermediate --output-file-prefix K562 --fastq-list /data/scratch/fastq_sheet.csv --ref-dir /data/scratch/hg38_altmaskedv2-cnv-hla-anchored.v8 --enable-sv true --qc-cross-cont-vcf /opt/edico/config/sample_cross_contamination_resource_hg38.vcf.gz --repeat-genotype-enable true --repeat-genotype-use-catalog default_plus_smn”. The resulting variant file (.vcf.gz) was then utilized for further preparation of input files (see details in Bioinformatics pipeline).

Oxford Nanopore direct RNA sequencing data for the cell lines K562 and HepG2 were downloaded from the Singapore Nanopore Expression Project (SG-NEx)^7^. The SG-Nex data was accessed on May 16, 2025 at registry.opendata.aws/sg-nex-data. Read data (.fastq) were mapped (minimap2 version 2.17 with “-ax splice -uf -k14” option) and used for the pipeline.

### Bioinformatics pipeline

Our pipeline requires three input files: [Input 1] A list of heterozygous putative splicing variants to be evaluated, [Input 2] A list of heterozygous variants used to distinguish between two alleles, referred to as “candidate allele-informative single nucleotide variants (candidate allele-informative SNVs)”, and [Input 3] Long-read data mapped against the reference genome (GRCh38 in this study).

[Input 1] When evaluating genome-wide splicing variants, we prepared the heterozygous splicing variants from short-read whole-genome sequencing data using snpEff annotation^13^ and SpliceAI (v1.3.1)^14^or solely based on SpliceAI scoring (for GM12878 (analyzing direct RNA sequencing), Control1, Patient1, and Patient2). After DRAGEN variant calling (described above), we used DRAGEN hard-filtered variants with a “PASS” status and selected heterozygous variants. When using both snpEff and SpliceAI, we first selected variants within splicing regions, defined as 1–3 bases into the exon from the exon-intron junction or 3–8 bases into the intron from the junction, plus branch point defined by snpEff (using the Nextflow-Sarek pipeline tool or by snpEff v5.2.1)^35,36^and then applied SpliceAI scoring to predict splicing variants. More specifically, we selected variants with a SpliceAI delta score of 0.5 point or higher as a default setting. We set each region of interest to the gene covered by nanopore long-reads, selecting the minimal and maximal coordinates predicted to be affected by SpliceAI.

[Input 2] For preparing candidate allele-informative SNVs, we selected SNVs with “PASS” and heterozygous state from the same sequencing data as in [Input 1].

[Input 3] We mapped each nanopore fastq reads to GRCh38 with minimap2 with “-ax splice -uf -k14” option.

Details and codes for preparation of input files are shown in Supplementary Note 5 and our github repository.

The following steps were performed for each splicing variant:

1. We defined the associated gene using SpliceAI annotation and assigned the genomic region to each splicing site.
2. We selected nanopore long reads spanning the splicing site and removed chimeric reads mapping both within the gene of interest and outside of it. Reads mapping outside the target gene were removed at the .bam file level using Picard (https://broadinstitute.github.io/picard/).
3. For each candidate allele-informative SNV within the gene region, we verified whether at least 10 nanopore long reads contained base (A/T/G/C) information at the SNV locus, regardless of allele identity. For processing allele-informative SNVs, we used the sam2tsv tool from jvarkit^15^.
4. To prevent mis-separation or mis-assignment, we filtered out nanopore long reads with base quality lower than 10 (90.0% reliable) at each allele-informative SNV using SamJdk from jvarkit^15^.
5. We then converted the bam reads, which had been separated into two alleles (pairs of .bam files), into paired fastq files for subsequent FLAIR calculation using the bedtools bamtofastq option^37^. Although it is preferable for long reads to be supported by two or more allele-informative SNVs for more reliable allele assignment, in practice, it is often difficult for a single long read to cover multiple allele-informative SNVs. Therefore, we allowed allele-separation or allele-assignment based on a single allele-informative SNV.
6. For long reads covering two or more allele-informative SNVs within the gene containing the splice site, we applied whatshap to generate haplotype-split reads^16^. After assigning haplotypes, these reads (.bam) were also converted to fastq files and processed further.
7. Finally, FLAIR (Full-Length Alternative Isoform analysis of RNA) calculations (flair_1.6.2: flair align, flair correct, flair collapse with the --support 4 and --no_gtf_end_adjustment options; flair quantify without using tpm option; plot_isoform_usage; and diff_iso_usage) were performed to generate isoform lists, diagrams, and usage graphs, along with Fisher’s exact test results. There calculations were performed on each paired long-read fastq files derived from.bam files separated by allele-informative SNVs. A statistically corrected p-value (using Bonferroni correction for the number of splicing variants examined by FLAIR) below 0.05 was considered significant for each pair; indicating an association with the splicing variant.

Parallelization was implemented throughout the pipeline using GNU parallel^26^. We implemented this pipeline in a LINUX (Ubuntu 22.04.1 LTS) workstation with the following hardware specificities: CPU, AMD Ryzen Threadripper 3990 × 64-Core Processor and Memory, 256Gbytes. GPU is physically installed but is not required in this pipeline per se. Because parallelization can be reduced as low as possible, one-core can work but is not practical. We recommend 32-core or more CPUs, while the memory size should be 32Gbytes at least. The time required for the preparation of mapped reads as well as other necessary files depends on the data themselves and is not included in this pipeline calculation time.

### Histogram and kernel density Estimation for considering read frequencies over read lengths

We used custom Python scripts to generate histograms and calculate kernel density estimation plots, utilizing pymc (version 5.13.1) and arviz (version 0.18.0).

### 5’ capped full-length cDNA nanopore sequencing (Cap trap RNA-seq, abbreviated as CTR-seq)

Library construction was conducted for CTR-seq similarly as in the previously published article^8^. Briefly, Total RNAs (5 ug) were isolated by RNeasy kit (QIAGEN). The cDNA synthesis was conducted with an RT primer (5-TTTTTTTTTTTTTTTTVN-3). Then full-length cDNAs were enriched with the Cap Trapper method^38^. 5’ linker was ligated, and second strand synthesis was conducted with KAPA HiFi HS mix (KAPA biosystems). The double-stranded cDNAs were amplified and subjected to Oxford Nanopore SQK-LSK110 library kit. Each library per individual was sequenced on an R10.3 flowcell (FLO-MIN111) in an Oxford Nanopore MINION sequencer. The raw data (.fast5) was basecalled by the Guppy basecaller (version 6.3.8) in superaccuracy mode (SUP).

### Nanopore long-read sequencing of amplified *PYGM* cDNA fragments

Sequencing of amplified *PYGM* cDNA fragments using primers (PYGM-long1520-2 F and PYGM-long1520-2R) listed in Supplementary Table 10 was performed with a nanopore MINION sequencer (Oxford Nanopore Technologies, UK) in conjunction with a Native Barcoding Kit 24 (SQK-NBD112.24). Briefly, PCR-amplified product from 10 ng of genomic DNA in a 50 ul reaction volume using Ex Premier Taq polymerase (TaKaRa bio, Japan) was purified with AMPureXP beads for subsequent barcoding according to the barcoding kit manual, followed by sequencing on an R9.4.1 flowcell in an Oxford Nanopore MINION sequencer. Demultiplexed and basecalled data (fastq files) were generated using the Guppy basecaller (version 6.5.7), followed by further analyses. Minimal base quality required at allele-informative SNVs was set to be 15.

### CRISPR/Cas9 enriched nanopore sequencing for haplotyping

For phasing the variants, we used CRISPR/Cas9-enriched nanopore sequencing of genomic DNA from the proband (II-1 in Supplementary Fig. 1a, corresponding to Patient2).

Briefly, crRNAs:

PYGM_left02_ext (Integrated DNA Technologies, USA, sequence: TCGAGACAAACCGTTCCCAT).

Predesigned Hs.Cas9.SF1.1.AB (Integrated DNA Technologies, USA, sequence: ATGCCAAGATTATGATCCGG).

were annealed with tracrRNA (Integrated DNA Technologies, USA) and complexed with S.p. HiFi Cas9 Nuclease V3 (Integrated DNA Technologies, USA) following the user manual. This complex was then used to specifically cut the genomic DNA, which had been dephosphorylated with alkaline phosphatase. Nanopore Y-shaped adapters were ligated to the DNA, and subsequent purification was performed using AMPureXP beads (BeckmanCoulter, USA). The prepared library was loaded onto an R9.4.1 nanopore MINION flowcell, according to the LSK-CS9109 kit instructions. 24 kb fragments generated by this sequencing were analyzed to reconstruct haplotypes (Supplementary Fig. 1d).

## Supplementary Information

Below is the link to the electronic supplementary material.

Supplementary Material 1

## Data Availability

The sequence variant identified in this study (NM_005609.3; c.1519-3T> G) is deposited in the ClinVar (https://www.ncbi.nlm.nih.gov/clinvar/) as ClinVar submitted record (SCV) accession number SCV005397959 (or Variation ID: 3387803). Implemented codes are deposited in github (https://github.com/ozakikokoro/ISAHESVAL).
