# Supplementary material for: Isoform analysis of heterozygous putative splicing variants at the allele level using nanopore long-read sequencing
Source: Sci Rep. 2025 Aug 8;15:29001. doi: 10.1038/s41598-025-14566-z (PMC12334610; doi:10.1038/s41598-025-14566-z)
Supplement: Supplementary file 1 — Supplementary Material 1 [file 41598_2025_14566_MOESM1_ESM.pdf]

Supplementary information

Contents

1. Supplementary Notes
Supplementary Note 1
Supplementary Note 2
Supplementary Note 3
Supplementary Note 4
Supplementary Note 5
Supplementary Note 6
Supplementary Note 7

2. Supplementary Figures
Supplementary Figure 1
Supplementary Figure 2
Supplementary Figure 3

3. Supplementary Tables
Supplementary Table 1
Supplementary Table 2
Supplementary Table 3
Supplementary Table 4
Supplementary Table 5
Supplementary Table 6
Supplementary Table 7
Supplementary Table 8
Supplementary Table 9
Supplementary Table 10

Supplementary Notes

Supplementary Note 1. Subject information for 5’ cap-trapping full-length cDNA sequencing

Control1: A 39-year-old healthy female, daughter of Patient1.

Patient1: A 72-year-old female with undiagnosed cerebellar ataxia.

Patient2: A 29-year-old male with glycogen storage disease type V (McArdle disease), also analyzed for *PYGM* amplicon sequencing.

Supplementary Note 2. Subject information for *PYGM* cDNA amplicon nanopore sequencing

Patient2: As described in 1.

Control1: As described in 1.

Control2: A healthy 36-year-old female.

Control3: A healthy 53-year-old male.

Supplementary Note 3. Clinical characteristics and genetic analysis of a 29-year-old man (Patient2) with Glycogen storage disease type V (McArdle disease)

Clinical characteristics: We managed a 29-year-old man who presented with muscle crumps or pain during exercise, which started when he was 12 years old. He had experienced episodes of elevated creatine kinase (CK) levels after exercise, reaching as high as 14978 IU/l, and sometimes noticed red to black urine. He had no family history of neuromuscular disease, though his mother occasionally experienced mild muscle cramps. His mother had eclampsia during his birth, leading to a caesarian section. At his first visit to our clinic at age 16, neurological findings were unremarkable, including normal muscle volume and strength. Laboratory tests showed elevated CK and transaminase levels following exercise, while other biochemical tests were unremarkable. The forearm ischemic exercise test showed a failure of lactate elevation in the blood, suggesting a glycogen metabolism deficiency. A muscle biopsy from the left quadriceps femoris revealed a loss of phosphorylase activity (Supplementary Fig. 1c), which is diagnostic of glycogen phosphorylase deficiency (McArdle disease; Glycogen Storage Disease type V). He was further characterized through subsequent genetic analyses.

Genetic analysis: Sanger sequencing of *PYGM* in the genomic DNA detected three heterozygous variants (an intronic variant (*PYGM*, NM_005609.3; c.1519-3T>G, chr11:64752507A>C (GRCh38)) within intron 12-13, located 3 bp upstream of the start of exon 13; a known pathogenic missense variant (c.347T>C, chr11:64758514A>G

(GRCh38), p.Leu116Pro) in exon 3; and a common variant (c.2412G>A, chr11:64746776C>T, p.;Arg804Arg). We analyzed 100 chromosomes from an in-house Japanese cohort of neurological disorders but without apparent primary muscular disease by Sanger sequencing and found that the variant (chr11:64752507A>C) was not detected. However, this variant was registered in the large Japanese public database, Tohoku Medical Mega Bank (ToMMo) 38KJPN, with an allele frequency 0.000103 (8 out of 77,444 alleles) and no homozygous individuals. It was not found in global gnomAD v3.1.2 database (76,156 genomes).

SpliceAI predicted that the novel intronic variant was highly likely to affect splicing (with delta scores of 0.98 for both acceptor gain and acceptor loss. In contrast, the allelic variant (c.1519-3T>C, chr11:64752507A>G (GRCh38)) was predicted not to affect splicing, with delta scores of 0 for acceptor gain, 0.03 for acceptor loss, 0 for both donor gain and loss.

Supplementary Note 4. Reverse transcription-polymerase chain reaction (RT-PCR) of *PYGM* cDNA fragments

From 500 ng of total RNA extracted from biopsied muscle (Trizol), Reverse transcription was performed using a mix of oligo-dT and random hexamer primers with the Perfect Real-time reverse transcription kit with gDNA eraser (TaKaRa bio, Japan). The amplification was done using the primers PYGM-long1520-2F and PYGM-long1520-2R. The amplified fragments were cloned into the Clontech Topo-vector pCRII Topo-Blunt in *E.coli* DH5α and Sanger sequenced using ABI3130xl sequencer (ThermoFisher, USA). Of the 18 clones were analyzed, 3 showed exon 13 skipping (Supplementary Fig. 3b), one showed an ectopic splicing acceptor site in exon 13, one showed both exon 13 skipping and an ectopic splicing acceptor site in exon 14, one showed skipping of exons 11-13 with an ectopic splicing acceptor site in exon 14, and one showed an ectopic splicing donor site in exon 17. The remaining 11 clones showed normal splicing (Supplement Fig. 3a).

Supplementary Note 5. Preparation of input files for the pipeline

(1) Here we describe typical commands and details for generation of two variant tables (one is the input putative heterozygous splicing variants, the other is variants used for searching for allele-informative SNVs). We recommend short-read whole genome sequencing for generation of raw data for this purpose. For the data in this work, we used DRAGEN Germline (v4.0.3) for mapping and variant-calling, as already written in the Method section. The ".hard-filtered.vcf.gz" and its index file ".vcf.gz.tbi" are needed for the following calculation. The script is also written in the github repository

(https://github.com/ozakikokoro/ISAHESVAL).

```
#-----(1) snpEff
input=/your_file_dir/your_sample.hard-filtered.vcf.gz
working_dir=$(dirname $input)
output_pre=$(basename $input)
output_file=${output_pre%.vcf.gz}_snpEff.ann.vcf.gz

# genome version that will be used for snpEff annotation
# which is downloaded already by a command like "snpEff download hg38"
genome_version=hg38

#conda activate snpeff (if you use snpEff in conda environment)
cd $working_dir
java -Xmx31g -jar /your_dir_for_snpEff/snpEff.jar ¥
-v -stats report.html $genome_version $input | bcftools view -O z -o ${working_dir}/${output_file}
#conda deactivate

#-----(2) bcftools basic filtering such as...
input2=/your_file_dir/your_sample.hard-filtered_snpEff.ann.vcf.gz
input2_short=${input2%.vcf.gz}
bcftools view ¥
-i 'FILTER=="PASS" && (GT=="0/1" || GT=="1/0" || GT=="0|1" || GT=="1|0" || GT=="1/2" || GT=="2/1" || GT=="1|2" || GT=="2|1")' ¥
-O z ¥
-o ${input2_short}.PASS_het.vcf.gz ¥
${input2}

tabix ${input2_short}.PASS_het.vcf.gz
#-----(3) bcftools splitting (converting compound heterozygous sites into heterozygous expression)
input3=/your_file_dir/your_sample.hard-filtered_snpEff.ann.PASS_het.vcf.gz
input3_short=${input3%.vcf.gz}
bcftools norm ¥
-m-any ¥
```

```
-O z ¥
-o ${input3_short}.split.vcf.gz ¥
${input3}

tabix ${input3_short}.split.vcf.gz
#-----(4) Only variants with annotations related to "splicing"
# IMPORTANT: This step(4) might be skipped and go to step(5).
input4=/your_file_dir/your_sample.hard-filtered_snpEff.ann.PASS_het.split.vcf.gz
bcftools view -h ${input4} > header.txt
bcftools view -H -O v ${input4} | grep "splice" > ${input4%.vcf.gz}.grep_splice.vcf_pre
cat header.txt ${input4%.vcf.gz}.grep_splice.vcf_pre >
${input4%.vcf.gz}.grep_splice.vcf
bgzip -c ${input4%.vcf.gz}.grep_splice.vcf > ${input4%.vcf.gz}.grep_splice.vcf.gz
tabix ${input4%.vcf.gz}.grep_splice.vcf.gz
rm ${input4%.vcf.gz}.grep_splice.vcf_pre header.txt

#-----(5) SpliceAI
input5=/your_file_dir/your_sample.hard-
filtered_snpEff.ann.PASS_het.split.grep_splice.vcf
# IMPORTANT: previous
# input4=/your_file_dir/your_sample.hard-filtered_snpEff.ann.PASS_het.split.vcf.gz
# might become an input in this step(5). Because the previous step (4) markedly reduce
the variants (by up to 1/1000!),
# especially when you implement CPU-version of SpliceAI, you should not at first time
skip the step(4), considering the vast computational time.
# Using input4 means splicing site prediction for genome-wide variants, while using
input5 means variants only in restricted regions
# (1-3 bases into the exon from the exon-intron junction or 3-8 bases into the intron from
the junction, plus branch point defined by snpEff).

reference=/your_references_dir/GRCh38_no_alt_analysis_set.fasta

spliceai -I ${input5} ¥
-O ${input5%.vcf}.spliceai.vcf ¥
-R ${reference} ¥
-A grch38
```

#-----(6) Filtering with spliceAI delta score threshold and converting .vcf into .csv file

You need three python scripts (split.py, compare.py, and filter.py) downloaded from this site into the same folder with a shell script file. filer.py will do a standard filtering of splicing variants with spliceAI's delta score of 0.5 or higher. If you prefer to use less stringent threshold, you can replace filter.py with the filter02.py, which will keep variants with delta score of 0.2 or higher. If you rather prefer more stringent threshold, you can replace filter.py with the filter08.py, which will keep variants with delta score 0.8 or higher. Here pandas module in python must be installed for calculation. The shell script file for these three python scripts (split.py, compare.py, and filter.py), for example, contains the following scripts:

```
# This is the script to convert spliceai_annotated vcf file (not bgzipped)
# into csv file (chr, coordinate, ref, alt, gene_symbol,start,end)
# Example: chr1,12345678,A,C,ATAD3A,12345600,12345700
# start,end are the coordinates used for samtools view, to restrict reads onto
# splicing variant and its affecting bases (see details in compare.py).

# Because spliceAI delta score of 0.5 point (acceptor increase|descrese | donor increase |
decrease) is a recommended threshold,
# we consider 0.5 point or higher score as a standard threshold for screening.

# This script requires split.py, filter.py, compare.py in the following folders.
# When you use delta score of 0.2 as threshold, you simply replace filter.py with
filter02.py

# Setting section here
input_file=/your_file_dir/your_sample.hard-
filtered_snpEff.ann.PASS_het.split.grep_splice.spliceai.vcf
input_dir=${input_file%/*}/
output_file=${input_file%.vcf}.converted.delta05.csv

# From here, script begins
splitting_python_script=/same_folder_as_this_script/split.py
filtering_python_script=/same_folder_as_this_script/filter.py
comparing_python_script=/same_folder_as_this_script/compare.py

mkdir tmp
```

```
cd ./tmp/

grep -v "^#" ${input_file} | grep "SpliceAI" | awk -F '¥t' '{print $1, $2, $3, $4, $5, $6, $7,
substr($8, index($8, "SpliceAI")), $9, $10}' > intermediate.txt
awk '{if ($8 ~ /,/) {split($8, a, ","); for (i in a) {sub(/^SpliceAI=/, "", a[i]); print
$1,$2,$4,$5,a[i]}} else {sub(/^SpliceAI=/, "", $8); print $1,$2,$4,$5,$8}}'
intermediate.txt ¥
> intermediate2.txt

# Set the input and output file paths
INPUT_FILE_split=intermediate2.txt
OUTPUT_FILE_split=intermediate3.txt

# Call the Python script with the input and output file paths as arguments
python ${splitting_python_script} $INPUT_FILE_split $OUTPUT_FILE_split
# This script makes a 13 columns data table.
# chr1 159927891 TAA TA IGSF9 . . . . . . . .
# chr1 159927891 TAA T IGSF9 0.00 0.01 0.00 0.00 -4 -7 -18 -16
# chr1 161599629 C G FCGR2C 0.98 0.13 0.00 0.00 1 30 1 -33

# Set the input and output file paths
INPUT_FILE_filter=intermediate3.txt
OUTPUT_FILE_filter=intermediate4.txt
# Call the Python script with the input and output file paths as arguments
python ${filtering_python_script} $INPUT_FILE_filter $OUTPUT_FILE_filter
# This script makes  13 columns data table
# filtering out spliceAI scores . . . .
# and retrieving  at least one of the four scores >=0.5.
#chr5 83353124 G A XRCC4 0.77 0.99 0.0 0.0 7 1 32 7
#chr5 96900192 A G ERAP2 0.0 0.0 0.0 0.51 -37 -3 20 -3
# Set the input and output file paths
INPUT_FILE_compare=intermediate4.txt
OUTPUT_FILE_compare=intermediate5.txt
# Call the Python script with the input and output file paths as arguments
python ${comparing_python_script} $INPUT_FILE_compare
$OUTPUT_FILE_compare
```

```
# This script makes 15 columns data table
#chr2 240950520 A G CROCC2 0.0 0.0 0.03 0.9 -42 -4 -10 -4 240950516 240950520
#chr3 14924000 A G FGD5 0.49 0.99 0.0 0.0 8 2 0 15 14924000 14924002
# $1,2,3,4,5,14,15 are the core data for subsequent analysis

# Use cut to extract columns 1-5, 14, and 15, using space as the delimiter
cut -d ' ' -f 1-5,14,15 "$OUTPUT_FILE_compare" > "$output_file"

# Replace space delimiter with comma = last step !
sed -i 's/ /,/g' "$output_file"

rm intermediate.txt intermediate2.txt intermediate3.txt intermediate4.txt intermediate5.txt
cd ../
rmdir tmp
```

(2) a variant file (.vcf) that is an intermediate file in the above scripts (annotation with snpEff -> basic filtering (heterozygous variant only)):
input3=/your_file_dir/your_sample.hard-filtered_snpEff.ann.PASS_het.vcf.gz
This file will be used as a variant list to search for candidate allele-informative SNVs.

(3) a nanopore mapped reads (.bam) that is mapped against the same version of the reference genome. For base-calling, SUP (superaccuracy) mode should be used for optimal base quality. Typically, mapping with minimap2 (with "-ax splice -uf -k14" option) is employed. Ensure that index file such as .bam.bai is also placed in the same folder with the .bam file. Transcript reads with any modalities (direct RNA sequencing, cDNA sequencing (amplified or not), cDNA targeted amplicon sequencing) can be an input.

Supplementary Note 6. Output folder/files
Result files are contained within a directory which is automatically created (e.g. out_20250425_1_v2_6), as follows (e.g. excerpt, produced by analysis of GM12878 direct RNA-seq data):
├── chr8_89983743_T_C_NBN_chr8_89935041_C_G (<-splicing variant, Gene Symbol, and its associated allele-informative SNV. This folder contains FLAIR result of long-reads covering the splicing variant and allele-informative SNV(s))

│ ├── allele1_flair.aligned.bam
│ ├── allele1_flair.aligned.bam.bai
│ ├── allele1_flair.aligned.bed
│ ├── allele1_flair_all_corrected.bed
│ ├── allele1_flair_all_inconsistent.bed
│ ├── allele2_flair.aligned.bam
│ ├── allele2_flair.aligned.bam.bai
│ ├── allele2_flair.aligned.bed
│ ├── allele2_flair_all_corrected.bed
│ ├── allele2_flair_all_inconsistent.bed
│ ├── concat_flair_all_corrected.sort.bed
│ ├── diff_iso_usage_result_filter.txt
│ ├── diff_iso_usage_result.txt
│ ├── flair.counts_matrix.tsv
│ ├── flair_ENSG00000104320.14_isoforms.png (<-diagram of isoforms)
│ ├── flair_ENSG00000104320.14_usage.png (<-isoform usage, colored in accordance with the above diagram of isoforms)
│ ├── flair.isoforms.bed
│ ├── flair.isoforms.fa
│ ├── flair.isoforms.gtf
│ └── manifest.tsv
├── chr8_89983743_T_C_NBN_selected.alt_2.chr8_89935041_G.NA12878-DirectRNA.pass.dedup.mm2.sort.bam (<-allele-separated reads)
├── chr8_89983743_T_C_NBN_selected.alt_2.chr8_89935041_G.NA12878-DirectRNA.pass.dedup.mm2.sort.bam.bai
├── chr8_89983743_T_C_NBN_selected.ref_2.chr8_89935041_C.NA12878-DirectRNA.pass.dedup.mm2.sort.bam (<-allele-separated reads)
├── chr8_89983743_T_C_NBN_selected.ref_2.chr8_89935041_C.NA12878-DirectRNA.pass.dedup.mm2.sort.bam.bai
(#Until here, files and folders are related to each splicing variant-allele-informative SNVs)
(#From here, following files are summary files for the run)
├── PN_minpvalue005_bampair_corrected.tsv (<-final table corrected by number of pairs (splicing variant and allele-informative SNV or haplotype associated))
├── PN_minpvalue005_spvar_corrected.tsv (<-final table corrected by number of splicing variants: main result)

├── PN_minpvalue005.tsv (<-final table showing splicing variant and associated allele-informative SNV or haplotype, but not corrected for multiple testing, filtered on the p-value 0.05 as the threshold for presence of isoform changes (FLAIR-calculated p-value)
├── PN_minpvalue.tsv (<-final table showing splicing variant and associated allele-informative SNV or haplotype, without no filtering on the p-value of presence of isoform changes (FLAIR-calculated p-value)
├── read_length_step1_fail_nonHLA.txt (<-experimental features for read-length calculation)
├── read_length_step1_fail.txt
├── read_length_step1_haplotyped_nonHLA.txt
├── read_length_step1_haplotyped.txt
├── read_length_step1_nonHLA.tsv
├── read_length_step1_pass_nonHLA.txt
├── read_length_step1_passORhaplotyped_nonHLA.txt
├── read_length_step1_passORhaplotyped.txt
├── read_length_step1_pass.txt
├── read_length_step1.tsv
├── read_length_step2_fail_nonHLA.txt
├── read_length_step2_fail.txt
├── read_length_step2_nonHLA.tsv
├── read_length_step2_pass_nonHLA.txt
├── read_length_step2_pass.txt
├── read_length_step2.tsv
└── Run_report.tsv (<-final summary for the calculation)

For quick understanding of each run, one may first look at Run_report.tsv and PN_minpvalue005_spvar_corrected.tsv. Then one may take a look at respective folder containing FLAIR-generated details for each splicing variant - allele-informative SNV pair. Only folders related to pairs with $p < 0.05$ isoform changes remain in the result folder ("out_yyyymmdd_v2_6").

Below is the example of the PN_minpvalue005_spvar_corrected.tsv:
id (chr and coordinate of splicing varinat and gene symbol) gene_symbol Ensemble_id chr coordinate ref alt chr_of_allele-informative_SNV coordinate_of_allele-informative_SNV Ref_of_allele-informative_SNV Alt_of_allele-informative_SNV Ref-covering_long-reads

Alt-covering_long-reads raw_p-value p-value_corrected_by_number_of_splicing_variants p-value_corrected_by_number_of_splicing_variant-allele-informative_SNV_combinations haplotype_information

chr12_109561243_C_T_MMAB MMAB ENSG00000139428.12 chr12 109561243 C T chr12 109573424 G T 37 34 1.48E-06 2.12E-04 1.52E-03 NA

chr12_109561243_C_T_MMAB MMAB ENSG00000139428.12 chr12 109561243 C T chr12 109573425 C T 42 31 7.14E-07 1.03E-04 7.38E-04 NA

chr12_109561243_C_T_MMAB MMAB ENSG00000139428.12 chr12 109561243 C T . . h1 h2 42 31 7.66E-11 1.10E-08 7.91E-08 spvar_is_on_1st_haplotype

chr19_4453238_C_T_UBXN6 UBXN6 ENSG00000167671.12 chr19 4453238 C T chr19 4454086 C T 119 117 5.25E-05 7.55E-03 5.42E-02 NA

chr21_44908184_T_C_ITGB2 ITGB2 ENSG00000160255.18 chr21 44908184 T C . . h1 h2 119 6 3.31E-09 4.77E-07 3.42E-06 haplotype_unavailable_to_spvar_nonexonic_or_indel

chr2_162279995_C_G_IFIH1 IFIH1 ENSG00000115267.9 chr2 162279995 C G chr2 162267541 C T 109 76 1.83E-38 2.64E-36 1.89E-35 NA

chr2_162279995_C_G_IFIH1 IFIH1 ENSG00000115267.9 chr2 162279995 C G chr2 162272314 T C 97 103 1.19E-49 1.72E-47 1.23E-46 NA

chr2_162279995_C_G_IFIH1 IFIH1 ENSG00000115267.9 chr2 162279995 C G . . h1 h2 1 0 2.19E-68 3.15E-66 2.26E-65 haplotype_unavailable_to_spvar_nonexonic_or_indel

P-value is the lowest p-value for isoform changes analyzed by FLAIR. When h1 h2 are written in the Ref/Alt of allele-informative SNV, the this line is information on whatshap created haplotype. When haplotype is created (that is, two or more allele-informative SNVs are available for this splicing variant), long-reads covering this haplotype region

are all analyzed by FLAIR.

Haplotype information for the splicing variant sometimes could not be calculated because the splicing variant was intronic (non-exonic) and not on the transcript long-reads, and is designated as "haplotype_unavailable_to_spvar_nonexonic_or_indel".

When haplotype information is available, to understand the relationship among the splicing variant and allele-informative SNVs, one has to look into associated phased variant file (for example, "chr12_109561243_C_T_MMAB_phased.vcf.gz"). In the ".phased.vcf.gz" file, which is created by whatshap, GT:PS tag is recorded, where phase 0|1 or 1|0 is written for each variant. For example, if the splicing variant and allele-informative SNV 1 have 1|0 and allele-informative SNV 2 has 0|1, then the splicing variant and allele-informative SNV1 on the same chromosome, while allele-informative SNV 2 is on the other chromosome. The example of splicing variant-associated ".phased.vcf.gz" is as follows:

#CHROM POS ID REF ALT QUAL FILTER INFO FORMAT NA12878

chr12 109561243(<-spvar) . C T 0 PASS KM=7.8;KFP=0;KFF=0;MTD=bwa_freebayes,bwa_gatk,bwa_platypus,isaac_strelka;ANN=T|intron_variant|MODIFIER|MMAB|ENSG00000139428|transcript|ENST00000545712.6|protein_coding|6/8|c.520-139G>A|||||| GT:PS 1|0:109561243

chr12 109561263 . G A 0 PASS KM=10;KFP=0;KFF=0;MTD=bwa_freebayes,bwa_gatk,bwa_platypus,isaac_strelka;ANN=A|intron_variant|MODIFIER|MMAB|ENSG00000139428|transcript|ENST00000545712.6|protein_coding|6/8|c.519+157C>T|||||| GT:PS 0|1:109561243

chr12 109573424(<-allele-i SNV_1) . G T 0 PASS KM=10.2;KFP=0;KFF=0;MTD=bwa_freebayes,bwa_gatk,bwa_platypus,isaac_strelka;ANN=T|synonymous_variant|LOW|MMAB|ENSG00000139428|transcript|ENST00000545712.6|protein_coding|1/9|c.57C>A|p.Arg19Arg|451/4438|57/753|19/250||,T|upstream_gene_variant|MODIFIER|MVK|ENSG00000110921|transcript|ENST00000228510.7|protein_coding||c.-1399G>T|||||388|,T|intragenic_variant|MODIFIER|MVK|ENSG00000110921|gene_variant|ENSG00000110921|||n.109573424G>T|||||| GT:PS 1|0:109561243

chr12 109573425(<-allele-i SNV_2) . C T 0 PASS KM=9.94;KFP=0;KFF=0;MTD=bwa_freebayes,bwa_gatk,bwa_platypus,isaac_strelka;ANN=T|missense_variant|MODERATE|MMAB|ENSG00000139428|transcript|ENST00000545712.6|protein_coding|1/9|c.56G>A|p.Arg19His|450/4438|56/753|19/250||,T|upstream_gene_variant|MODIFIER|MVK|ENSG00000110921|transcript|ENST00000228510.7|protein_coding||c.-1398C>T|||||387|,T|intragenic_variant|MODIFIER|MVK|ENSG00000110921|gene_variant|ENSG00000110921|||n.109573425C>T|||||| GT:PS 1|0:109561243

Supplementary Note 7. Comparison of variant calling by DRAGEN Germline on whole genome sequencing data with illumina Hiseq and MGI DNBSEQ-G400

We downloaded whole genome sequencing data (fastq.gz files) from the following source and mapped to GRCh38 and conducted variant-calling as follows:

Illumina Hiseq paired end PCR-free 40x GIAB WGS data retrieved from demo data in illumina basespace titled "HG001 and Chinese Trio (HG005, HG006 and HG007) downloaded from https://github.com/genome-in-a-bottle/giab_data_indexes and downsampled to ~40x" was used. HG001(GM12878) data was loaded onto DRAGEN Germline v4.0.3 and analyzed with the same options as in K562 and HepG2 (described in the Methods section), except for downsampling to 30x coverage.

For MGITech DNBSEQ-G400, we accessed demo data site on the https://en.mgi-tech.com/resource/demo/ping_id/1/ying_id/4/du_id/1 and downloaded the fastq.gz files from https://ftp.cngb.org/pub/CNSA/data1/CNP0000466/CNS0094977/CNX0058578/CNR0077391/. This data, according to the site, was the data by the library kit MGIEasy PCR-Free DNA library Prep Set and sequencing in a DNBSEQ-G400 on NA12878. After downloading, header was reconstructed to apply to illumina-based applications. Briefly, index sequence NNNNNN was added to the header columns after converting MGI type to illumina type by bgi2illumina. After that, filtering reads that have the same length for sequence and quality was conducted and abnormal reads without headers starting with "@" were removed. After these filtering, the reads data was processed with DRAGEN Germline v4.0.3 using the same setting with illumina data and downsampled to 30x similarly.

The hard-filtered.vcf.gz files for both were filtered for "PASS" in quality and compared with each other for intersection. For calculation of F1 score, variants identified by illumina were treated as the true variants. Observed number of variants and calculated values are shown in Supplementary Table 9.

Supplementary Figure 1. McArdle disease patient (Patient2) harboring a novel intronic variant and a known missense pathogenic variant

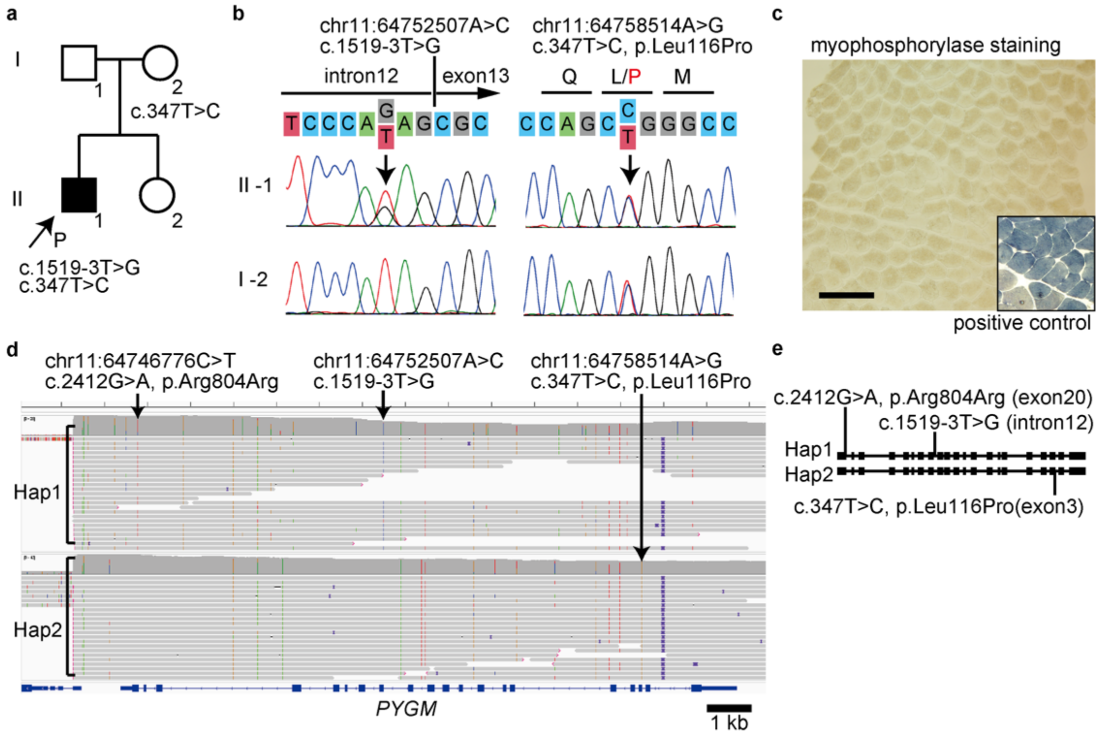

a, The family pedigree of the patient with McArdle disease (Patient2). The arrow with “p” indicates the proband. Circles indicate females, squares indicate males, and a black-filled symbol indicate an individual of McArdle disease.

b, Electropherograms from Sanger sequencing of *PYGM* performed in the mother (I-2) and the proband (II-1). The proband (II-1) harbored a novel splicing pathogenic variant (c.1519-3T>G (NM_005609.3), chr11:64752507A>C (GRCh38)) and a known missense pathogenic variant (c.347T>C, p.Leu116Pro, chr11:64758514A>G). Whole-genome sequencing and other genomic and transcript analyses were performed on the proband (II-1).

c, Muscle biopsy stained for myophosphorylase activity showed a severe loss of activity in the proband (II-1), in contrast to the staining control.

d and e, Haplotyping using CRISPR/Cas9-enriched nanopore sequencing of genomic DNA from the proband (II-1) showed that the novel splicing variant (c.1519-3T>G) and the missense pathogenic variant (c.347T>C) were in trans. Thus, the proband was a compound heterozygote for the two variants. He also carried a common synonymous variant (c.2412G>A). Detailed information is provided also in Supplementary Note 3 and 4.

Supplementary Figure 2. Distribution of read frequency by read length, with or without significantly different isoform changes between alleles

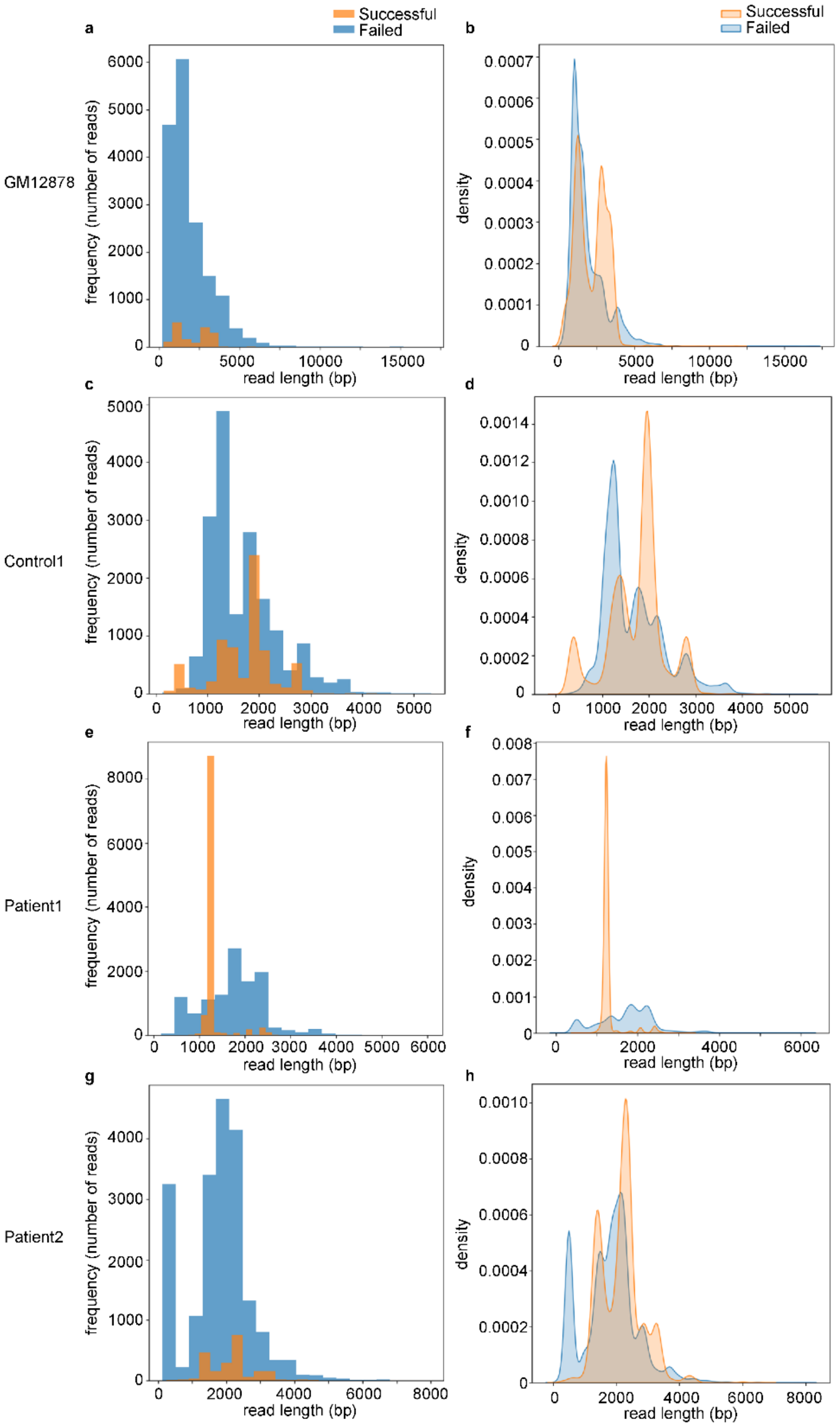

Histograms of read frequency (number of reads by read length) for GM12878, Control1, Patient1, and Patient2 (a, c, e, and g, respectively), and kernel density estimation plots (a type of estimated probability curve based on the data) for GM12878, Control1, Patient1, and Patient2 (b, d, f, and h, respectively).

Supplementary Figure 3. Exon 13 skipping of *PYGM* detected in biopsied muscle from a patient with McArdle disease

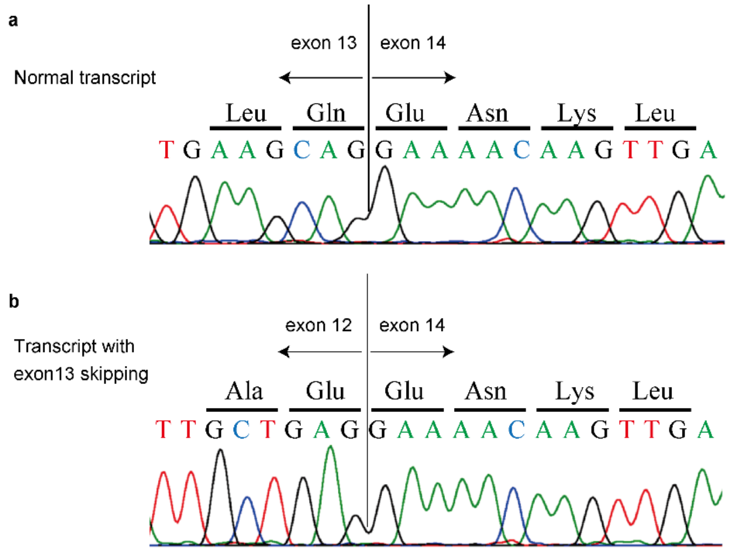

RT-PCR product of *PYGM* from total RNA of biopsied muscle in the McArdle disease patient (Patient2) was cloned in *E.coli* (DH5 α). Of the 18 clones analyzed, 3 showed exon 13 skipping (b), and 11 showed normal splicing (a). Detailed information is provided in Supplementary Note 4.

Supplementary Table 1. List of identified genes and heterozygous putative splicing variants associated with allele-specific changes in isoform content by direct RNA sequencing in GM12878

Gene Symbol	Ensemble ID	Chr	Position	Ref	Alt	Lowest corrected p value between alleles
DRAM2	ENSG00000156171.15	chr1	111139496	C	T	1.734E-16
TPP1	ENSG00000166340.17	chr11	6617154	C	T	1.368E-03
TOR1AIP1	ENSG00000143337.19	chr1	179889309	G	A	3.923E-10
MMAB	ENSG00000139428.12	chr12	109561243	C	T	1.103E-08
UBXN6	ENSG00000167671.12	chr19	4453238	C	T	7.555E-03
ITGB2	ENSG00000160255.18	chr21	44908184	T	C	4.765E-07
IFIH1	ENSG00000115267.9	chr2	162279995	C	G	3.148E-66
HLA-A	ENSG00000206503.13	chr6	29944633	G	A	0.000E+00
HLA-DPA1	ENSG00000231389.7	chr6	33070214	C	G	1.198E-19
HLA-DPA1	ENSG00000231389.7	chr6	33070256	G	A	1.198E-19
HLA-DPA1	ENSG00000231389.7	chr6	33070276	C	T	1.198E-19
HLA-DPB1	ENSG00000223865.11	chr6	33080684	T	A	3.490E-81
HLA-DPB1	ENSG00000223865.11	chr6	33085905	G	A	8.393E-81
HLA-DPB1	ENSG00000223865.11	chr6	33086378	C	T	6.126E-81

Input number of putative splicing variants: 401 (SpliceAI delta score threshold = 0.5). Minimum number (threshold setting) of reads covering splicing variants and allele-informative SNV(s): 10. Chr: Chromosome, Ref: Reference base, Alt: Alternative base.

Supplementary Table 2. List of identified genes and heterozygous putative splicing variants associated with allele-specific changes in isoform content by direct RNA sequencing in cancer cell line K562

Gene Symbol	Ensemble ID	Chr	Position	Ref	Alt	Lowest corrected p value between alleles
GIPC1	ENSG00000123159.16	chr19	14478566	C	T	4.90E-02
RIPK2	ENSG00000104312.8	chr8	89784139	GGTA	G	2.50E-02

Input number of putative splicing variants: 70 (SpliceAI delta score threshold = 0.5). Minimum number (threshold setting) of reads covering splicing variants and allele-informative SNV(s): 10. Chr: Chromosome, Ref: Reference base, Alt: Alternative base.

Supplementary Table 3. List of identified genes and heterozygous putative splicing variants associated with allele-specific changes in isoform content by direct RNA sequencing in cancer cell line HepG2

Gene Symbol	Ensemble ID	Chr	Position	Ref	Alt	Lowest corrected p value between alleles
ULK3	ENSG00000140474.14	chr15	74837752	T	C	1.27E-12
XRCC4	ENSG00000152422.16	chr5	83353124	G	A	9.90E-06

Input number of putative splicing variants: 101 (SpliceAI delta score threshold = 0.5). Minimum number (threshold setting) of reads covering splicing variants and allele-informative SNV(s): 10. Chr: Chromosome, Ref: Reference base, Alt: Alternative base.

Supplementary Table 4. Read statistics for full-length 5' cap-trapped and poly-A captured whole transcriptome nanopore long-read sequencing (CTR-seq) of three subjects

Subject	reads	bases	Median read length (bp)	N50 length (bp)	Median read quality
Control1	8,286,998	19,760,731,505	1,608.00	3,171.00	13.24
Patient1	9,579,385	17,012,330,352	1,317.00	2,136.00	13.27
Patient2	6,761,892	16,473,629,333	1,721.00	3,200.00	13.30

Read statistics created by PycoQC report for Pass reads.

Supplementary Table 5. List of identified genes and heterozygous putative splicing variants associated with allele-specific changes in isoform content by CTR-seq in Control1

Gene Symbol	Ensemble ID	Chr	Position	Ref	Alt	Lowest corrected p value between alleles
DRAM2	ENSG00000156171.15	chr1	111139496	C	T	1.380E-48
TCHP	ENSG00000139437.18	chr12	109904793	G	A	9.748E-13
SPSB2	ENSG00000111671.10	chr12	6872998	C	T	1.923E-16
GSKIP	ENSG00000100744.15	chr14	96379755	G	A	4.995E-59
CTSH	ENSG00000103811.18	chr15	78944772	TC	T	2.192E-02
RPS28	ENSG00000233927.5	chr19	8321817	G	A	3.555E-10
ITGB2	ENSG00000160255.18	chr21	44908184	T	C	3.016E-07
NUP50	ENSG00000093000.19	chr22	45164760	T	C	3.849E-02
ANKRD36	ENSG00000135976.20	chr2	97167605	G	A	1.145E-02
BTN3A2	ENSG00000186470.14	chr6	26368051	G	A	1.076E-08
HLA-A	ENSG00000206503.13	chr6	29943484	C	G	2.476E-83
HLA-DRA	ENSG00000204287.14	chr6	32443258	A	C	4.354E-03
HLA-DRB5	ENSG00000198502.6	chr6	32520438	A	C	4.016E-253
HLA-DRB5	ENSG00000198502.6	chr6	32530124	C	T	1.409E-251
HLA-DRB1	ENSG00000196126.11	chr6	32581554	C	A	1.916E-29
HLA-DQA1	ENSG00000196735.13	chr6	32643157	A	G	8.401E-66
HLA-DQB1	ENSG00000179344.16	chr6	32660883	T	C	1.794E-244
HLA-DPA1	ENSG00000231389.7	chr6	33070214	C	G	4.074E-44
HLA-DPA1	ENSG00000231389.7	chr6	33070256	G	A	4.074E-44
HLA-DPA1	ENSG00000231389.7	chr6	33070276	C	T	4.074E-44
HLA-DPB1	ENSG00000223865.11	chr6	33080684	T	A	0.000E+00
HLA-DPB1	ENSG00000223865.11	chr6	33085905	G	A	0.000E+00
HLA-DPB1	ENSG00000223865.11	chr6	33086378	C	T	0.000E+00
CTSB	ENSG00000164733.22	chr8	11848168	C	G	4.412E-03
VPS28	ENSG00000160948.14	chr8	144424955	G	A	4.292E-02

Input number of putative splicing variants: 420 (SpliceAI delta score threshold = 0.5). Minimum number (threshold setting) of reads covering splicing variants and allele-informative SNV(s): 10. Chr: Chromosome, Ref: Reference base, Alt: Alternative base.

Supplementary Table 6. List of identified genes and heterozygous putative splicing variants associated with allele-specific changes in isoform content by CTR-seq in Patient1

Gene Symbol	Ensemble ID	Chr	Position	Ref	Alt	Lowest corrected p value between alleles
RBM23	ENSG00000100461.18	chr14	22905226	G	A	3.620E-04
CD37	ENSG00000104894.12	chr19	49339567	G	A	3.844E-09
XRCC4	ENSG00000152422.16	chr5	83353124	G	A	1.351E-02
HLA-A	ENSG00000206503.13	chr6	29943484	C	G	3.558E-73
HLA-DQB1	ENSG00000179344.16	chr6	32660883	T	C	7.281E-183
HLA-DPA1	ENSG00000231389.7	chr6	33070256	G	A	3.510E-04
HLA-DPB1	ENSG00000223865.11	chr6	33080684	T	A	1.339E-227

Input number of putative splicing variants: 389 (SpliceAI delta score threshold = 0.5). Minimum number (threshold setting) of reads covering splicing variants and allele-informative SNV(s): 10. Chr: Chromosome, Ref: Reference base, Alt: Alternative base.

Supplementary Table 7. List of identified genes and heterozygous putative splicing variants associated with allele-specific changes in isoform content by CTR-seq in Patient2

Gene Symbol	Ensemble ID	Chr	Position	Ref	Alt	Lowest corrected p value between alleles
TOR1AIP1	ENSG00000143337.19	chr1	179889309	G	A	2.511E-50
RNASEL	ENSG00000135828.11	chr1	182581225	C	T	6.555E-04
GSKIP	ENSG00000100744.15	chr14	96379755	G	A	2.194E-25
IL16	ENSG00000172349.17	chr15	81308110	T	G	2.479E-03
PDXDC1	ENSG00000179889.19	chr16	15008848	G	A	3.744E-02
ANKRD36	ENSG00000135976.20	chr2	97167605	G	A	1.772E-04
ANKRD36	ENSG00000135976.20	chr2	97179736	A	G	1.399E-04
ULK4	ENSG00000168038.11	chr3	41915974	T	G	9.244E-09
BST1	ENSG00000109743.11	chr4	15722935	G	A	8.045E-76
HLA-DRA	ENSG00000204287.14	chr6	32443258	A	C	3.440E-04
HLA-DRB1	ENSG00000196126.11	chr6	32589642	C	G	2.319E-03
HLA-DQB1	ENSG00000179344.16	chr6	32660883	T	C	1.247E-109
HLA-DPA1	ENSG00000231389.7	chr6	33070214	C	G	5.688E-10
HLA-DPA1	ENSG00000231389.7	chr6	33070276	C	T	5.688E-10
IRF5	ENSG00000128604.20	chr7	128938247	T	G	4.862E-07

Input number of putative splicing variants: 457 (SpliceAI delta score threshold = 0.5). Minimum number (threshold setting) of reads covering splicing variants and allele-informative SNV(s): 10. Chr: Chromosome, Ref: Reference base, Alt: Alternative base.

Supplementary Table 8. Typical calculation time in the pipeline on the direct RNA sequencing of GM12878

Delta score threshold	Number of putative splicing variants as input	real	user	sys	user + sys
0.2	2425	160.4 hours	1299.2 hours	391.7 hours	1690.9 hours
0.5	401	22.6 hours	192.8 hours	67.4 hours	260.2 hours
0.8	104	3.9 hours	26.6 hours	9.9 hours	36.5 hours

Supplementary Table 9. Number of variants called by DRAGEN Germline (v4.0.3) on whole genome sequence data at coverage 30x by illumina HiSeq and MGITech DNBSEQ-G400

Class (short variant)	Unique to illumina HiSeq	Unique to MGI DNBSEQ-G400	Common	Precision	Recall	F1 score
all	170622	187781	4673255	0.96137017	0.96477574	0.96306994
snps	110999	94488	3824711	0.97589099	0.97179696	0.97383967
indels	60773	94593	852476	0.90012027	0.93345407	0.91648417

Supplementary Table 10. List of primers used in this study

Utility	Primer name	Sequence (5' to 3' direction)
Genomic PCR for Sanger sequencing exon 1	PYGM_exon1_F	GGCTGGAGGCAGTGCTGAGG
Exon 1	PYGM_exon1_R	GCAGCCACTTAAGTCAAGATCG
Exon 2-4	PYGM_exon2_F	TGGGCCTGGCTGAGTGTTGG
Exon 2-4	PYGM_exon4_R	CCAGAGATGATAAACAAGTGGG
Exon 5	PYGM_exon5_F	GAGCTCCTGACTTATACTTGG
Exon 5	PYGM_exon5_R	CTCTCTGAGCCTCAGCATCC
Exon 6-7	PYGM_exon6_F	TAAAGCCTTGAGTCCCAGCC
Exon 6-7	PYGM_exon7_R	TAGGGCACCAGCAAGTGTCC
Exon 8	PYGM_exon8_F	ACCCACCGCAGCTTTAGGCCATGC
Exon 8	PYGM_exon8_R	AGAGGCCTAGCACACACTGTCC
Exon 9	PYGM_exon9_F	CATAGGGCAGTGACCATAGCATGG
Exon 9	PYGM_exon9_R	CTCCCACGCTCCCAAACTGG
Exon 10	PYGM_exon10_F	GGGATGCGTAGTGTGTGAGG
Exon 10	PYGM_exon10_R	CGACTCCCAGGCCCAGACTGG
Exon 11	PYGM_exon11_F	TTCCTGGGTCTGGTTCTAGC
Exon 11	PYGM_exon11_R	GGGCTTCTGTGTGACAGAGG
Exon 12	PYGM_exon12_F	CCCTTTCAGCAAGACACCAGG
Exon 12	PYGM_exon12_R	GTGAACCACCTACACGACCATACC
Exon 13	PYGM_exon13_F	GCTTGGCTGACCTGGAAATGG
Exon 13	PYGM_exon13_R	CACGCCTGACCCAGACATCTGG
Exon 14	PYGM_exon14_F	ACTGGAGTGTGGACTGTAGG
Exon 14	PYGM_exon14_R	AAGTCCAAAGGAGATGTTGG
Exon 15-16	PYGM_exon15_F	CTCTGTCAGGAGCTACTACC
Exon 15-16	PYGM_exon16_R	AAGTATCCCAGGAAGAGACG
Exon 18-20	PYGM_exon18_F	GACCTGGGTTTTGACCCTGG
Exon 18-20	PYGM_exon20_R	AGAGATCTAACTCCAGTACC
RT-PCR forward	PYGM-long1520-2F	GACAAGGCGTGGGATGTG
RT-PCR reverse	PYGM-long1520-2R	CCTCTGCATGAGGTGCTG
